# Rethinking the digital divide in health: a critical interpretive synthesis of research literature

**DOI:** 10.3389/fdgth.2025.1683565

**Published:** 2026-01-15

**Authors:** Meghan Bradway, Bo Wang, Henriette Lauvhaug Nybakke, Stine Agnete Ingebrigtsen, Kari Dyb, Eirin Rødseth

**Affiliations:** 1 Norwegian Centre for E-Health Research, University Hospital of North Norway, Tromsø, Norway; 2 Faculty of Health Sciences and Social Care, Molde University College, Molde, Norway; 3 Department of Social Sciences, University of Tromsø—The Arctic University of Norway, Tromsø, Norway

**Keywords:** critical interpretive synthesis, digital divide, digital health, digital inequalities, eHealth, health technology, mHealth

## Abstract

**Background:**

The digital divide in health has rapidly expanded during and after the COVID-19 pandemic, with fragmented understanding and an unclear implementation process, for the formal integration of digital health into the healthcare system, which challenges actionable policy development.

**Methods:**

This critical interpretive synthesis (CIS) of the literature aimed to capture the complexity of the digital divide in health. This began with a scoping review of literature published between 2013 and 2023 describing the digital divide in health within the WHO's European Region, in Web of Science, Medline (via Ovid), PsycInfo (via Ovid), and Sociological Abstract (via ProQuest). Three sets of two reviewers independently conducted the selection, and all contributed to the synthesis process.

**Results:**

Of 4,967 original articles identified, 49 articles were included for review. Results revealed a synthesizing argument that the digital divide should be considered as more of a dynamic, entangled, and reciprocal collection of “areas” of phenomenon affecting service users, rather than “levels”. Results describe the three synthetic constructs that describe this synthesizing argument.

**Conclusion:**

Findings suggest that digital health solutions should respectfully consider the pace of human healing, long-term user engagement and adaptability. We call for the importance of inter- and multidisciplinary collaboration to ensure effective and context-sensitive implementation in future studies.

## Introduction

The digital divided is broadly understood as a phenomenon in which groups are differentiated by characteristics such as socioeconomic background, age, and gender which contribute to challenges in terms of access, knowledge, competence, and costs of digital recourses (1). While the concept of the digital divide occurs in every aspect of life, in this paper we focus on its impact on the health sector. This phenomenon is slowly growing beneath the expanding surface of healthcare digitalization, especially during and after the COVID-19 pandemic. The result is an exacerbation of existing social inequalities and polarization of society (1, 2). However, the concept of digital inequality in healthcare has evolved beyond the traditional definition of “digital divide,” which originally centered on access to digital technology and the Internet (3, 4). It now encompasses a wider scope, including the second-level digital divide (skills and uses) and third-level digital divide (outcome and autonomy) (3, 5, 6).

### What do we know about the digital divide in health?

There are several terms that are commonly used to describe the digital divide, e.g., digital inequality, inequity, exclusion, poverty or disparity. It is considered a “super determinant of health” due to the digitization of healthcare services and information, and the internet's influence on other determinants of health, e.g., socioeconomic status, access to fresh food and education, even before the COVID-19 pandemic (7). For example, in 2019, 10.42% of European Union households did not have internet access (8), and only 53.18% of 18–64 year old internet-users sought health information online (9).

Najeh Aissaoui (2022) described a comprehensive landscape of factors that contribute to the digital divide. Their review revealed that most literature referred to at least one of these levels: Level 1: Access and availability of internet and computers, Level 2: motivation and use, and Level 3: Outcomes or effects of using technology. The first concept of the digital divide was limited to the Level 1. However, the complexity of the digital divide paralleled the development of technology and more recently has included the second and third levels to reflect technologies’ increasing availability, diversity of functionalities and potential uses, and a greater understanding of how people are impacted by it (10).

Solutions have been proposed to “close”, “bridge” or “narrow” the digital divide gap, spanning across the individual, community and societal levels. Some examples are to increase information flow amongst healthcare providers, develop culturally safe digital solutions, and partner with community organizations (11, 12). However, Najeh Aissaoui (2022) describes that the imbalance of research between the three levels of digital divide, lack of global assessment and of actionable theoretical framework for the third level digital divide combine to challenge these solutions and perpetuate the divide (10).

### What does policy need to act on health research knowledge?

The terms “evidence based” policy and healthcare practice are often used and make it sound as though the translation of research knowledge to practice in the health field is natural or an effective established process (13). However, a discouraging reality of health research is that most outcomes are less actionable or go unnoticed (13–15). As such, there are multiple divides that need to be considered to address the digital divide in health (15). One is the divide between research knowledge and implementation processes. Another is the divide between implementation and policy generation and action. Communication between these areas is hindered by knowledge that is siloed, slow to produce and uptake, in different vernaculars, and unactionable. In fact, “Research is only as useful as potential users perceive it to be, irrespective of its methodological rigour or its findings’ power” (16).

Resources that span these divides in the form of channels, people or processes that translate and iteratively develop knowledge between sides, are needed to make them actionable by downstream actors. Oliver et al. (2014) highlighted that the formatting and tailoring of research findings to policy makers, e.g., in terms of language, tailoring of information and dissemination, significantly impacted the uptake of research results by policy makers (17). Common literature reviews form curated knowledge that is diverse and are presented in lists that segregate types of information, which that make it difficult for policy makers to generate a consensus on its clinical effectiveness, or even its intended use (18).

### Presented paper

There is a clear need to re-think the format and context of results regarding the digital divide in health before effective solutions can be developed. In this paper, we acknowledge the need for actionable information to enable (a) researchers to identify specific next steps based on gaps in knowledge, (b) those developing digital health technologies to incorporate more factors and more representative populations into their development to ensure more relevant technologies, and (c) healthcare authorities and policy makers to understand and address the greater complexity of factors that affect integration of technology. This review is part of a larger project, *Tackling social inequalities in health with the use of e-health and telemedicine solutions,* which focused on disease specific telemedicine models in Poland and Norway (19). Tasked with exploring potential reasons for digital inequalities that may arise during the parent project, the authors of the presented paper intend to understand the current situation of the digital divide for similar health systems within Europe*.* The use of a Critical Interpretive Literature Synthesis (CIS) will allow for a more contextualized, conceptually accessible, and thereby more actionable, understanding of the digital divide in health. This is just one piece of the puzzle toward finding solutions to the digital divide in health through a greater and more accurate understanding of the phenomenon (20).

### Research questions

We aim to identify a comprehensive understanding of how the literature describes health-related digital divide for individuals by answering the following research questions:

RQ1. Who experiences the digital divide in health?
RQ2. What type of health technology are they “divided” from?
RQ3. Why are they experiencing the digital divide?

## Methods

We intended to perform a scoping review of the literature to provide an update on the concept of the digital divide in health. The scoping review process followed the Preferred Reporting Items for Systematic Reviews and Meta-Analyses (PRISMA) checklist (21) (Supplementary File S1). However, after initial data extraction, it did not seem accurate to solely report the categories of themes and subthemes. Instead, patterns and connections between reported information and categories pushed us toward a CIS method for literature reviews. We report both a summarized version of the scoping review results and full results from the CIS review to provide equally useful, yet different, forms of knowledge garnered from this review of the literature.

### Critical interpretive synthesis

The CIS is a relatively new approach of knowledge curation that can serve to format and contextualize research outcomes within the needs and agendas of health-related policy makers (22). Such agendas are exemplified by the WHO's Evidence-informed Policy Network (23) and other experts in the field of health research knowledge transfer who refer to needs including contextualization in terms of: currently relevant concepts including determinants of health, health and technology inequalities, and socio-political and cultural environments in which the digital health solutions in question are meant to be used (15, 24).

As described by Dixon-Woods et al. (2006), a CIS is a form of interpretive review that allows for reflexive, iterative and flexible approaches to describing our current knowledge of the digital divide provided by scientific literature (22). This was especially appropriate for this review given that digital health technologies affect and are affected by a complex web of actions and interactions between individuals, industry, healthcare system, and society (22). The ultimate purpose of a CIS is to develop concepts and contribute to theory based on contrast and comparison across literature via interpretive methods (25, 26). The two common outputs are synthetic constructs and synthesizing argument based on an initial thematic analysis. While there is no current standard for reporting interpretive reviews, we followed the suggested list of criteria for reporting by Depraetere et al. (27): data extraction, synthesizing argument, inclusion of various methods, quality appraisal, two-staged sampling process, and broad searching strategy (27). We summarize our method below and provide details of our protocol for the review, analysis and synthesis of the literature in Supplementary File S2

#### Search strategy

Two systematic searches were conducted to ensure all relevant up-to-date articles were included. The first (performed February 22, 2023) for literature published between 2013 and 2023, while the second (performed January 23, 2025) covered literature published between 2023 and 2025. Both searched Web of Science, Medline (via Ovid), PsycInfo (via Ovid), and Sociological Abstract (via ProQuest) after consultation with a research librarian. Searches were limited to title, abstract, and keywords and followed the PICO search strategy:

Population: those who experience inequalities related to the use of digital health technologies
Intervention: digital health technologies
Comparison: not applicable
Outcomes: factors or reasons that contribute to a person or groups’ experience of the digital divide

#### Selection criteria and sampling

Using Rayyan software, four pairs of reviewers independently screened abstracts and titles (28). Studies were included if they were: published between the years 2013 and 2025, in English, Danish, Norwegian or Swedish languages. Studies also must have been performed within the World Health Organization's European Region and contain original empirical data. Studies were only included if they described healthcare service users (>18 years) who experience the digital divide. Studies were excluded if they involved people under the age of 18 years, were conducted outside of the WHO's European region, or focused on COVID-19. Protocols, reviews, commentaries, and gray literature (Websites, tweets, and blogs) were also excluded. Reasons for exclusion were detailed for each article by the reviewers (HLN, SAI, MB). Authors’ previous experience with reviews and studies about digital health technology (MB), knowledge of the digital health field (all authors) and sociological knowledge of inequality (HLN, SAI, KD) informed inclusion criteria.

After abstracts were screened, reviewers in each group met to resolve disputes before all four groups achieved consensus and derived a final list of studies for full-text review.

The full-texts articles were reviewed by three authors: MB (quantitative and mixed method studies), HLN and SAI (qualitative studies). Any uncertainties were discussed and decided upon by all authors.

Because some articles were deemed appropriate to be included in the review yet did not meet all specific inclusion criteria, a second review was performed. Only articles that included information that answered all three research questions were discussed amongst reviewers and ultimately included if agreement was reached.

#### Quality assessment

To evaluate the quality of the include articles, we employed the Mixed Methods Appraisal Tool (MMAT) (29). This tool was designed for the appraisal stage of systematic mixed studies reviews, comprised of quantitative, qualitative, and mixed method studies (30). MMAT is based on criteria that are specific to the method used and includes the following: the suitability and rigor of the methods used, control of confounding factors, minimization of selection bias, and consideration of limitations (30). Additionally, we required details of ethical approval and informed consent. The MMAT scoring was conducted by two researchers (MB and HLN) independently, followed by mutual discussion. When no agreement could be reached regarding the assessment, a third researcher (WB) was consulted.

#### Data extraction

MB created a data-extraction template for use by all reviewers including the following: date of publication, country where study was performed, health condition, study type, intervention or type of technology described, target group recruited, reason for being considered target group and experiencing the digital divide, and sub-groups identified. Text from the articles were exported verbatim into a Microsoft Excel file to minimize bias.

#### Analysis and synthesis

We were informed by the stepwise deductive induction (SDI) approach to a meta-ethnographic analysis and adapted this approach to fit a CIS. This was well suited for a CIS because it allowed us to include different study designs and their resulting data, as well as guide the development of theory (31). We understand that while this method is meant for raw empirical data, we treated the article text, i.e., authors' interpretations of their findings, as empirical data. The six steps of SDI are:

Taking raw data
Generating empirical data
Inductively processing data into codes
Taking the code-structured data and grouping codes or categories
Coding groups to develop a concept model
Discussing concepts to generate theory (which is not always possible) (31)

These steps can be re-framed into two stages of the CIS methodology whereby the first four steps represent the thematic analysis of raw data followed by the fifth and sixth steps which produce synthetic constructs and final synthesizing argument(s) (Supplementary File S2).

All data was translated into qualitative data in order to perform this abductive thematic analysis akin to the meta-ethnographic method of reciprocal translational analysis (RTA) (2, 32, 33). We used a inductive approach to code the empirical data and group codes into categories. Because the theory that there were three levels of the digital divide was already well documented, we used an deductive approach to consider the three levels as our three themes. Refutational analysis provided the foundation for the “critical” part of the critical interpretive synthesis, which came from questioning the accuracy of this 3-level digital divide model given the new evidence. This involved identifying emergent patterns or relationships between the “actors” in the existing digital divide model, i.e., target group members, societal context and technology, and the influence of study design and personal situations of study participants. These patterns formed the synthetic constructs of the CIS approach. HLN and SAI performed this process using NVivo 13 software and MB used Microsoft Excel.

All authors of the presented paper reviewed these synthetic constructs, or characteristics of the digital divide, to provide their input from their perspectives in psychological health (BW), sociology (KD, HLN, SAI), digital health development (MB), and social anthropology (ER) with the intention of preventing bias from any one tradition of scientific inquiry (Supplementary File S2).

## Results

**Figure 1:**
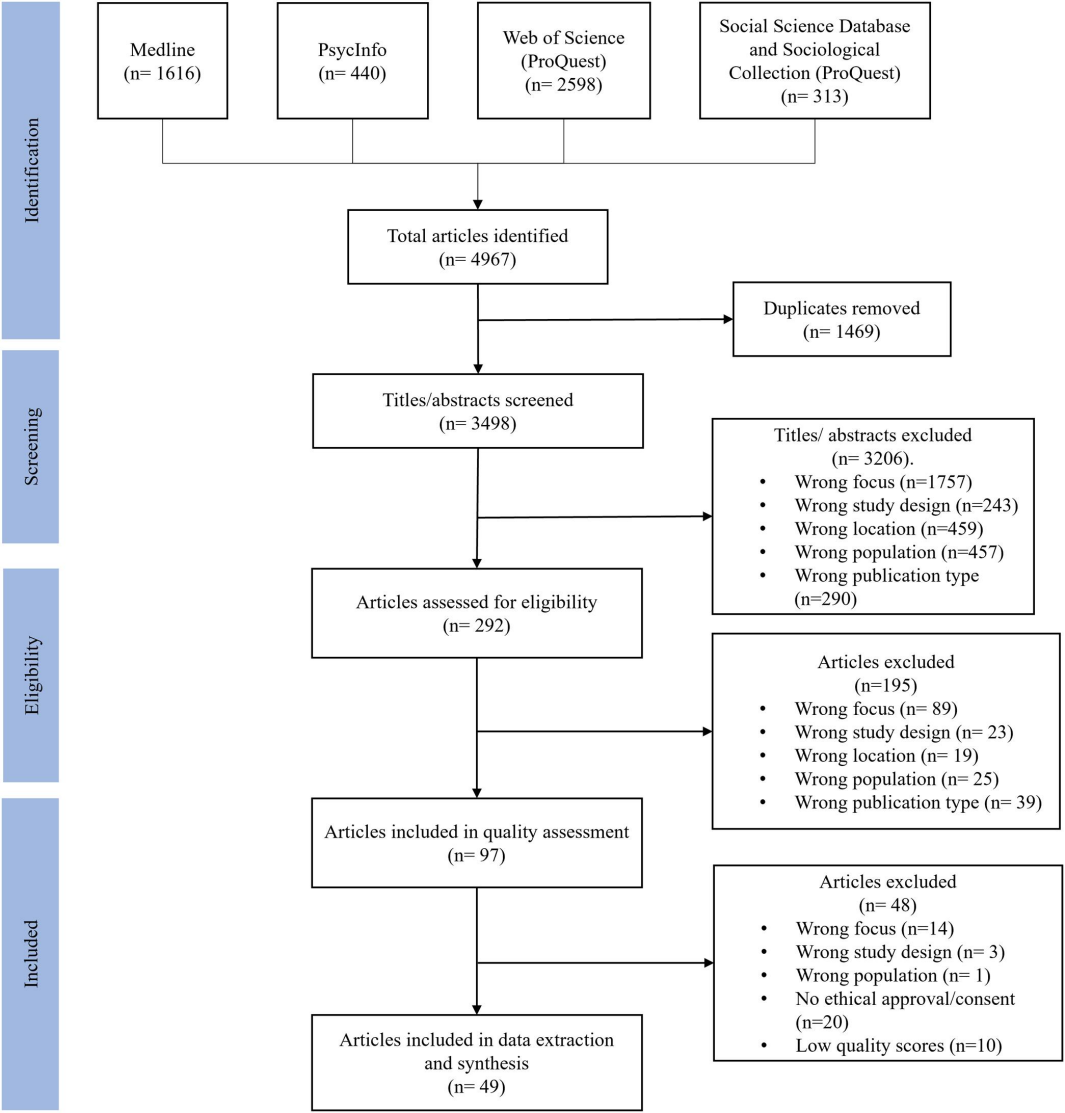
PRISMA flow diagram adapted to the CIS.

The literature search produced 4,967 records for review (Figure 1). After removing 1,461 duplicates and non-relevant papers, 292 full papers were assessed. Finally, 195 papers were excluded if they did not answer all research questions and 49 papers were included in the data extraction and synthesis.

Of the 49 articles included for synthesis, 24 were quantitative, 3 were mixed methods and 22 were qualitative. Studies were completed in Denmark (*n* = 4), Finland (*n* = 7), Germany (*n* = 3), Hungary (*n* = 2), Ireland (*n* = 1), Netherlands (*n* = 1), Norway (*n* = 4), Sweden (*n* = 8), the UK (*n* = 15), and two studies included more than one country (UK, Spain, Italy *n* = 1, and France, Germany, Belgium *n* = 1). Most studies (*n* = 20) did not focus on individuals with a specific health condition, whereas individuals with the following conditions were focused on by one article each: chronic kidney disease, multiple chronic diseases (hypertension, diabetes, COPD), chronic pain, dermatological conditions, frailty, neurodivergence, cardiometabolic conditions, substance use, and pregnancy. The remaining 20 focused on individuals with the following health conditions: Cancer (*n* = 3), Cognitive impairment (*n* = 4), mental health conditions (*n* = 6), neurological conditions (*n* = 2), and Type 1 and/or 2 diabetes (*n* = 3). An overview of included articles is described below (Table 1).

**Table 1:** Summary descriptions of included articles.

Authors [reference]	Year	Country	Study type	Health condition studied
Hardy, A. et al. (34)	2022	UK	Quant	Distressing paranoia
Powell, J. and Deetjen, U. (35)	2019	UK	Mixed	N/A
Poduval, S. et al. (36)	2018	UK	Quant	Type 2 Diabetes
Neves, A.L. et al. (37)	2021	UK	Quant	N/A
Bol, N. et al. (38)	2018	Netherlands	Quant	N/A
Heponiemi, T. et al. (39)	2020	Finland	Quant	N/A
Heponiemi, T. et al. (40)	2023	Finland	Quant	N/A
Radó, N. et al. (41)	2022	Hungary	Quant	N/A
Holmberg, C. et al. (42)	2022	Sweden	Quant	Psychotic disorders
Villadsen, S.F. et al. (43)	2020	Denmark	Quant	Pregnancy
Puaschitz, N.G. et al. (44)	2021	Norway	Quant	Dementia
Papp-Zipernovszky, O. et a (45)	2021	Hungary	Quant	N/A
Mattsson, S. et al. (46)	2017	Sweden	Quant	Cancer
Quittschalle, J. et al. (47)	2020	Germany	Quant	N/A
Buchert, U. et al. (48)	2023	Finland	Mixed	N/A
Poli, A. et al. (49)	2021	Sweden	Quant	Outpatient surgery
Bruno, E. et al. (50)	2020	UK	Quant	Epilepsy
Rantanen, T. et al. (51)	2022	Finland	Quant	N/A
Tetri, B. and Juujärvi, S. (52)	2022	Finland	Quant	Mental health
Paccoud, L. et al. (53)	2021	France, Germany, Belgium	Quant	N/A
Rantanen, T. et al. (54)	2021	Finland	Quant	N/A
Dahlhausen, F. et al. (55)	2022	Germany	Qual	N/A
Korn, S. et al. (56)	2022	Germany	Quant	Outpatient surgery
Hansen, A.H. et al. (57)	2019	Norway	Quant	Type 1 and Type 2 Diabetes
Bhargava, S. et al. (58)	2019	Norway	Qual	Breast cancer (screening)
Nymberg, V.M. et al. (59)	2019	Sweden	Qual	Chronic diseases (hypertension, diabetes, COPD)
Simblett, S. et al. (60)	2019	UK, Spain, Italy	Qual	Depression
O'Reilly, P.M. et al. (61)	2022	Ireland	Qual	Chronic pain
Safarov, N (62)	2021	Finland	Qual	N/A
Greer, B. et al. (63)	2019	UK	Qual	Mental illness
Vaportzis, E. et al. (64)	2017	UK	Qual	N/A
Middle, R. and Welch, L. (65)	2022	UK	Qual	Severe mental illness
Ong, B.N. and Sanders, S. (66)	2021	UK	Qual	N/A
Vereenooghe, L. et al. (67)	2017	UK	Qual	Intellectual disability
Landgren, S. and Cajander, Å. (68)	2021	Sweden	Qual	N/A
Husebø, A.M.L. (69)	2021	Norway	Qual	Colorectal Cancer
Buckingham, S.A. et al. (70)	2022	UK	Qual	N/A
Mathiesen, A.S. et al. (71)	2017	Denmark	Qual	Type 2 Diabetes
Chadwick, H. et al. (72)	2024	UK	Qual	Chronic kidney disease
Pettersson, L. et al. (73)	2023	Sweden	Quant	Impairment
Ramjee, S. et al. (74)	2023	UK	Quant	Dermatology
Turnbull, J. et al. (75)	2024	UK	Quant	N/A
Davoody, N. et al. (76)	2023	Sweden	Qual	Aphasia due to stroke
Canet-Vélez, O. et al. (77)	2023	Spain	Qual	Frailty
Löthberg, M. et al. (78)	2024	Sweden	Mixed	Neurodivergence
Tarp, K. et al. (79)	2024	Denmark	Qual	Alcohol use
Pacheco Lorenzo, M. et al. (80)	2023	Spain	Qual	Cognitive impairment
Gybel Jensen, C. et al. (81)	2024	Demark	Qual	Neurological conditions
Ramasawmy, M. et al. (82)	2024	UK	Qual	Cardiometabolic disease

**Figure 2:**
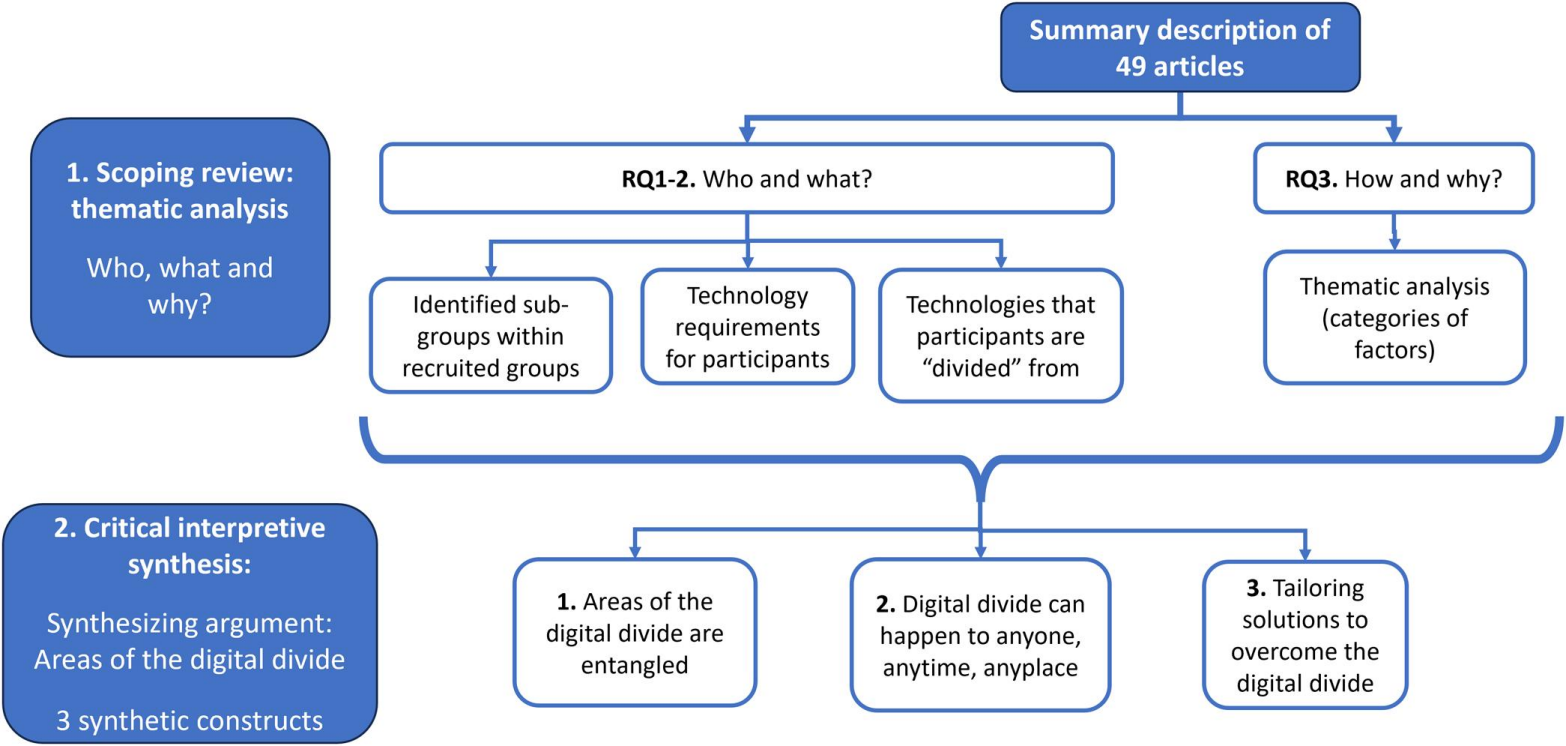
Organization of results section.

The remaining results are divided into three main sections: in the first two sections, we summarize findings related to our research questions (Supplementary File S3). In the third section, we present the results of the interpretive synthesis of the literature (Figure 2).

### RQ1 and RQ2: who experienced the digital divide—and from what?

While intended recruitment focused on a group in the methods, there were often distinct sub-groups described in the results sections. Groups were commonly identified based on gender, age, social marginalization, lack of previous experience, and socioeconomic status. Within these groups, some studies reported that anyone could experience issues like discontinuing use or lacking trust in technology, while others specified challenges amongst a representative population (38). For example, a study involving patients with distressing paranoia from community mental health services (34), found that ethnic minorities, older people, black people, women, and people living in the inner-city (London) experienced the following: lack/limited tech knowledge, confidence, self-efficacy (literacy), or skills (lack training, skills are out of date/no longer relevant, or are too slow to keep pace). Whereas in the same study, minority ethnic groups and men experienced less engagement or interest. Men specifically reported discontinued use (including technology does not support needs over time), one-time registration with no further use, and lower perceived usefulness over time (34). In Supplementary File S3, Supplementary Table S1 provides a comprehensive list of identified groups and their specific challenges.

Secondly, describing a group or person as “digitally divided” requires context and what they are excluded from. Some studies had specific technology-related requirements for participants (Supplementary File S3, Supplementary Table S2). Most studies did not require use of technology for inclusion of participants (*n* = 28), whereas others required prior use (*n* = 5), ownership (*n* = 3), expert knowledge (*n* = 1), completion of digital survey (*n* = 8), and adequate computer skills (*n* = 1). Six studies (63, 65, 68, 73, 79, 80) only included those who lacked experience with the digital technology. One study highlighted that older age “Increased the likelihood of being non-screened, non-recruited or declining participation due to technology-related barriers, rather than age itself being the barrier” (49).

We also noted which technologies were described or tested in each article (Supplementary File S3, Supplementary Table S2). The most common technologies mentioned were: Online or digital formal health and social care services (*n* = 23) (36, 37, 39, 40, 42, 43, 48, 51–55, 57–59, 62, 66–68, 75, 76, 78, 81), internet or digital technology for general health purposes (*n* = 17) (35, 41, 45–47, 56, 61, 63–65, 69–74, 82), Mobile health apps, wearables or sensors (*n* = 8) (34, 38, 44, 49, 50, 60, 77, 80) and blended care (*n* = 1) (79).

### RQ3: how and why do people experience the digital divide?

We identified three overarching themes, or three “levels” of the digital divide: access and availability (1st level), motivation and use (2nd level), and outcomes or effects of using technology (3rd level).

Five sub-themes, or categories, were identified: *Infrastructure (societal and healthcare system), Personal, Technology, Providers or healthcare personnel.* The third level of the digital divide only described three categories: *Healthcare system, Personal* and *Technology*. All categories were further divided into factors or more specific reasons that contribute to the digital divide (Supplementary File S3, Supplementary Table S3). The most common reasons related to access and availability of technology (1st level digital divide) were, as expected, *Accessibility or availability* of technology, including the internet, related to the category *Infrastructure (societal and healthcare system)* (34–36, 39–41, 43–46, 48, 53, 54, 56, 58, 61, 66, 70–74, 76–80, 82). The most common reasons that affected motivation and use (2nd level digital divide) were *Acceptance*, including disinterest in, distrust, motivation, choice (*n* = 34) (34, 36, 39–41, 43, 45, 48–52, 54, 56, 58–63, 65–69, 71–73, 77–82), *Engagement* with technology, including suboptimal or discontinued use (*n* = 34) (34, 36, 39–41, 43, 49–51, 53–57, 59–61, 63–66, 68, 70–75, 77–82), and *Competency and/or capacity,* including knowledge and skills for use, competing responsibilities, negative beliefs about abilities (*n* = 33) (34, 39–42, 49, 50, 52–61, 65–74, 76–79, 81, 82), all related to the category *Personal*. The most common reason for not receiving the benefits of digital health or experiencing negative outcomes (3rd level digital divide) were related to *Personal impact*, including intrusiveness of technology, negative self-perceptions, general personal dangers, negative personal experiences (*n* = 17) (39, 40, 46, 48–51, 58, 61, 65, 69–72, 78, 81, 82) under the category *Technology*, and *Societal relationships*, including discrimination, stigma, social isolation connectedness and reliance on others (*n* = 14) (39, 40, 45, 48, 49, 51, 67, 69, 71, 77, 78, 80–82) under the category *Personal*. The use of technology could also negatively influence a person's Competency and/or capacity (i.e., Personal category) relative to the quickly evolving nature of digital health, e.g., not being able to keep up with the necessary skills (54, 56, 72, 77, 80, 81), the inability to balance the time and energy necessary to use technology with other priorities in life (77, 82), or limiting the ability of groups to learn and retain new practical life skills, which often required in-person contact (78, 79). Participants in several studies, believed that the use of remote health support would negatively affect the relationship and communication with healthcare providers (60, 61, 66, 67, 70, 72, 77–80), which could lead to poorer quality care (61, 66, 67, 71, 78), resulting in poorer health outcomes (60, 67, 72, 78, 79).

### Critical interpretive synthesis results

**Figure 3:**
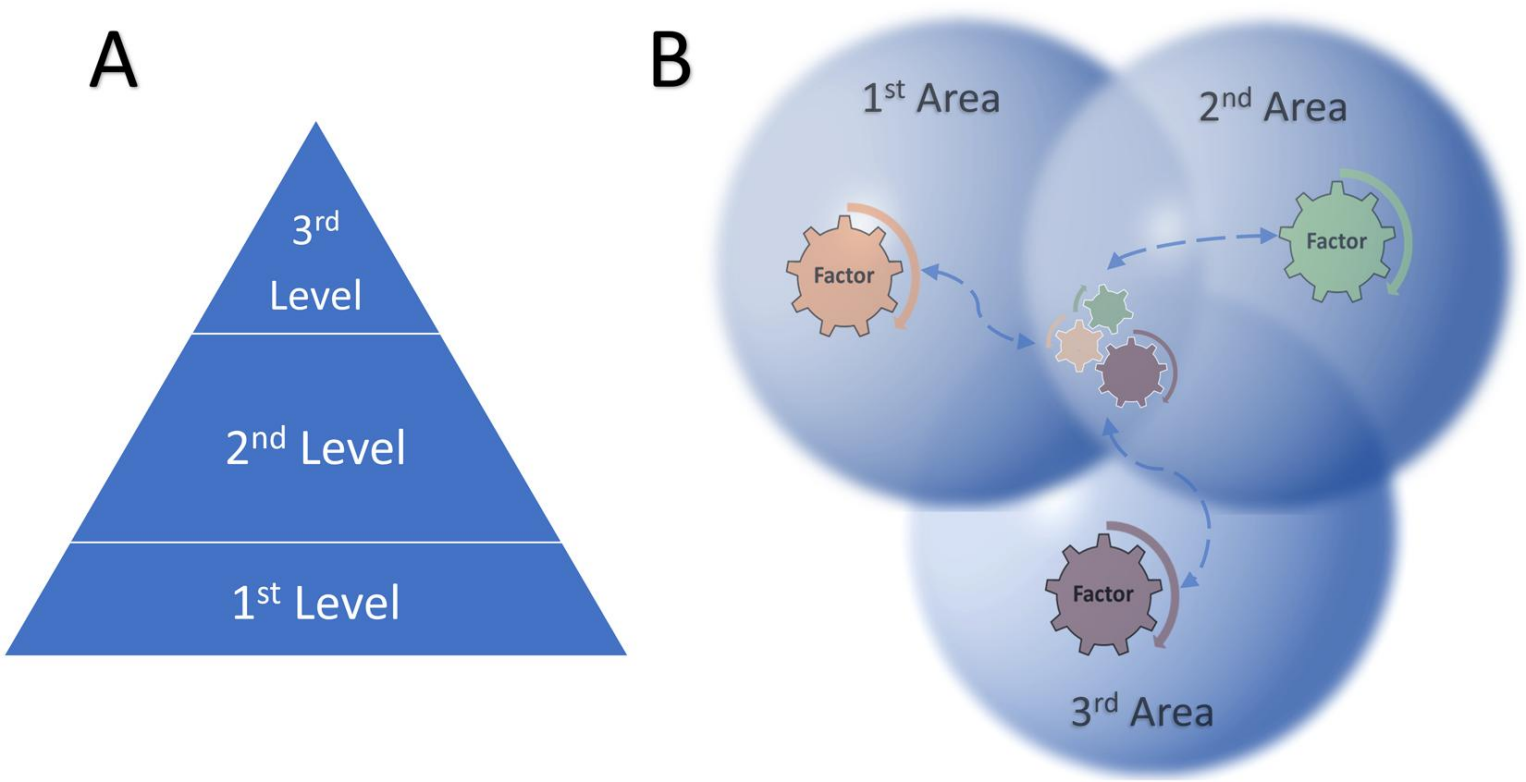
Illustration comparing relationship between “levels” of the digital divide **(A)** vs. the more accurate “Areas” of the digital divide **(B)**.

The main outcome, or the synthesizing argument, of the CIS review was a change in perception that the digital divide is working in “Areas” and not “Levels”. When describing the active working relationship between factors, levels are less accurate as they imply that there is a unidirectional, and at best—bi-directional- working relationship. Areas more accurately describe the context and interactions between categories, factors and individuals or groups. This term allows for a more entangled and dynamic conceptualization (Figure 3). Therefore, we will refer to “areas” of the digital divide for the remainder of the text.

The interpretive synthesis of the literature revealed three synthetic constructs of the digital divide. These appear as numbered sections below: (1) Areas of the digital divide are entangled, (2) Digital divide can happen to anyone, anytime, anywhere, (3) Tailoring solutions can overcome the digital divide. It is important to note that authors of these articles described the context in which the digital divide occurred and therefore most articles represented more than one emergent characteristic.

#### Areas of the digital divide are entangled, inextricably linked, cyclical, reciprocal, and simultaneous

The scope and mechanisms of reasons for experiencing the digital divide are complex and evolving. By assessing the context in which factors existed, we noted that the areas of digital divide were intersectional and non-linear. In other words, they are inextricably linked while also being equally cyclical, reciprocal, and simultaneous. By this we mean that, due to the number and diversity of factors, changes or activities can be happening in any one of them at any point in time, which can affect other factors, either increasing or decreasing one's challenges with health technology.

First—factors do not act independently. Most studies explained relationships between factors and that multiple factors could occur simultaneously. So, if we imagine the factors like cogs in a machine (Figure 2. Illustration of levels vs. areas), it becomes clear how they can combine to produce the digital divide. For example, for older individuals suffering from chronic pain, engagement with an eHealth program for chronic pain management was influenced by several factors in the 2nd divide: (a) concern that the intervention would not provide the relevant support *(Technology)*, (b) lack of supporting information from intervention providers (*Providers and healthcare personnel)*, (c) concern about their own ability to continue the intervention after the study and without formal support *(Personal)* and (d) difficulty navigating the digital program due to a combination of physical symptoms, age, depression and poor technology functionalities *(Personal* and *Technology)* (61) (Supplementary File S3, Supplementary Table S3).

Some studies described factors as interacting indirectly and/or directly (51, 55). In one such study, incarcerated people were asked about their relationships with digital health care and social welfare services. General internet self-efficacy was found to indirectly impact the use of digital health services by mediating the relationship between general self-efficacy and perceived control. Interestingly, general self-efficacy also had a direct impact on perceived control, but was, in fact, weaker than general internet self-efficacy's indirect impact on digital health services (51).

In a qualitative study exploring perceived relationships between access to digital therapeutic technologies (DTx) and patient use, expert vendors of DTx reported that healthcare providers' decision not to prescribe DTx to a patient directly limited patient access. Healthcare providers' were directly impacted, and patients were indirectly impacted by, by insufficient information, hurdles related to protocol, limited motivation, lack of time and lack of financial incentives for healthcare providers (55). Exploring further, pharmaceutical companies and insurance providers indirectly affected patients’ access by limiting such information and financial incentives for healthcare providers. However, they could also directly impact patients' access by providing -or limiting—information to patients so they could request DTx from their providers (55).

Second- continuing with the analogy of cogs in a machine—the digital divide can occur because of one factor (one malfunctioning gear), even when other factors for inclusion were present. In a study of wearable seizure trackers, people with uncontrolled seizures were diligent about wearing the device. However, because they forgot to pair or charge their device, 19.8% of the total number of seizures were not captured (8/30 participants). Others (36.7%) required additional training from intervention providers or support to correct use (50). Other studies described that, while individuals may have the access, motivation and skills necessary to use digital health tools, healthcare providers could act as a barrier. Providers may have (a) low acceptance of health technology based on their beliefs about and trust in its suitability for clinical care (67), or (b) low competency or capacity to use it based on individual and healthcare providers’ concern of the potential negative impact on resources as well as providers’ low digital literacy or training (55), as well as (c) challenges with reimbursement (55), and (d) concern for the risk of compromising quality of care (59, 60, 67, 68, 71, 78). One study in particular reported that participants with alcohol use disorder were opposed to the use of digital or remote healthcare services because it distanced them from treatment providers who served to keep patients accountable, and therefore, decrease patients' accountability toward treatment (79).

Third—our machine cogs are not static. Several studies described that the factors related to individuals' use of digital health technology often varied over time, correlating with their health conditions, or even discontinued all together. Discontinued use was reported in studies when participants, for example, had skills but lacked motivation to continue engaging with a patient portal to improve self-management as soon as their health improved (37). Similarly, participants living with aphasia due to stroke in a co-design study revealed that, while the technology was not suitable for the current level of their disease progression, they believed it would have been helpful had it been introduced earlier in their rehabilitation, when they experienced the most need for communication support and greater cognitive capacity (76). Another example was a study in which digital mental health service users reported being less inclined to use mobile health technologies when experiencing significant symptoms of depression due to lack of motivation and “emotional resources”, despite otherwise following recommended use (60).

Fourth—social inequality and the digital divide in health are inextricably linked. This is evident in several of the categories and factors listed within all areas of the digital divide, e.g., social background (social position, health status, personal support and resources). The relationship in which the non-digital meets the digital divide is explained by Helsper's corresponding fields model, which was referenced by several articles (35, 39, 40, 48, 51, 54, 62, 63, 65). The model argues that a person's resources offline affect their ability and use of digital tools. The resulting digital divide reinforces factors related to social inequality. For example, those who were not given the opportunity to learn digital skills in their working years often lacked the skills to use digital health after retirement (72). Others reported that they feared being passed over for promotion due to lack of digital skills, which is included as a 3rd area digital divide factor (45). As such, factors of social inequality and any area of the digital divide build upon one another to create a cycle of inequity.

One study categorized four domains of social resources within Helsper's corresponding fields model, which can be directly linked to the digital divide: personal (e.g., physical and mental health), cultural (e.g., gender or religious affiliation), economic (e.g., poverty or low education), and social (e.g., social participation). The study itself demonstrated the link between personal, economic and social inequalities offline and lower perceived personal, economic, and social online benefits (3rd area factors), which were only significant when the mediators of poorer skills, access and negative attitudes about online services (1st and 2nd) were included (*p* < .001) (40). Negative social outcomes of digital health use described in other studies included social isolation (48, 71, 77, 78). Cultural illegitimacy was cited as a social barrier to the use of digital devices when people experiencing lower socio-economic status believed that the use of digital devices was a privilege to which they did not have the right (53). Health-related factors could also represent social inequities that contribute to the digital divide related to access (48, 68) or skills and use (41, 42, 44–46, 54, 59–61, 65–73, 77, 78, 80–82). The ability of those with dementia to access digital assistive devices was dependent upon support from informal carers. Specifically, determinants of access included the carers’ co-morbidities, physical function, and age in addition to the person with dementia's age, gender and proper use of the telephone (44). The combination of social position and resources was presented in a study of Russian-speaking older migrants who moved to Finland. Migration away from established social networks meant that these individuals must rebuild their social network, and relatively quickly, to participate in the new society and digital services. As a result of such migration, their low income, unemployment, low digital literacy and age contributed to lack of confidence and non-use of health technologies. Because they reside in such a digitally advanced country, such non-use could contribute to an even greater social exclusion. Even respondents who were tech-savvy, educated and proficient in the Finnish language had to use a third-sector organization to use digital social insurance services because the technical, medical and bureaucratic language of the system were too advanced. In fact, the authors described digital services as “rigid to the point of being merciless” (48).

#### Digital divide can happen to anyone, anytime, anywhere

The previous characteristic reveals several examples of groups who commonly experience the digital divide due to expected inequities as resources and health challenges. However, the literature also introduces other factors that, depending on the context, can affect anyone.

One observation was that technology is not made for everyone, with two interpretations of the phrase “made for”. Firstly, technology is not universally designed, but instead primarily made for the capable or digitally engaged. Lack of relevance or suitability of the technologies for groups with specific health needs consistently appeared in the literature (44, 51, 54, 56, 66–70, 72, 76–82). The concept of then “digital inverse care law” was introduced to describe the situation in which those who could benefit the most from digital health support are not capable to use technology themselves and do not receive the necessary support to achieve these benefits (61). Several studies mentioned that participants were not able to complete digital health tasks successfully without help, e.g., setting up the security measures, or constant instructions (39, 40, 52, 53, 65, 68, 71, 76, 81, 82). An interesting variation of this concept was “the proxy effect”. The proxy effect describes situations in which people who cannot or are uncomfortable with using technology themselves ask others to perform the task for them (49, 52, 72, 82). The downside of this, however, was the risk that those needing the digital health technologies would become dependent on others and not learn how to use it themselves (39, 40, 49, 53, 67, 69). Secondly, not all technology is desired by everyone. Both interpretations were evident in a study of selective participation, i.e., non-recruitment, non-screening, or declining. Choice was a common reason for not engaging with digital health technologies, which also related to lack of relevance, lack of desire, distrust, or satisfaction with current healthcare treatment (34, 36, 39–41, 43, 45, 48–52, 54, 56, 58–63, 65–69, 71–73, 77–82). Fear of dependency on technology—going so far as to call it an “addiction” (82) and others including scepticism toward remote management, as described above (60, 61), technology's lack of empathy (51, 60, 69, 72, 79), especially in times of mental health crisis (78), or concern that technology would make them less physically active or lazy (69, 82) also contributed to participants' choice to avoid digital health. Authors argued that the consequence of such choice or of not being recruited by digital health studies was “incorrectly inform[ed] digitalization policies and practice” (49).

The concept of preference becomes more complicated if you consider that one person's choice to use one type of health technology over another can mean that they are “divided” from one while “included” by another (56, 69, 80), or when they discontinue using a specific digital health device (a factor also mentioned above) because behaviour changes were in competition with previous “unhealthy” health habits (61).

Studies also demonstrate that just because someone “belongs” to a certain group previously reported as a digitally included group, does not mean that person is immune to the digital divide in some way. While most studies argued that younger age was associated with digital inclusion, there are combinations of situations and technologies that create digital divide for young adult populations. For example, young people who were marginalized, based on education and socioeconomic status, become digitally divided because they need assistance to use digital health care services. In a study comparing generational cohorts (e.g., Baby boomers and Gen Z), authors present that while younger generations (college-aged) are confident in using the internet and have higher digital health literacy compared to older generations, college-aged students were not confident when it came to making health decisions independently from digital information (45). Even fear of perceived negative judgement, self-perception or misunderstanding was strong enough to result in non-use of digital health technologies, regardless of their membership in this typically digitally included group based on their age (78). Also, being a man is generally associated with greater acceptance of use of technology. However, men were dissuaded from using digital mental health services due to stigma (54).

#### Tailoring solutions to overcome the digital divide

The results of this interpretive synthesis also demonstrate that the digital divide does not necessarily mean digital exclusion—someone can have difficulty using all functionalities of a technology or using it as it was intended but still have access to, be able to use and gain benefit from certain functionalities or applied uses. Studies demonstrated that considering individuals, their situations and potential to tailor technologies could prove successful in addressing factors of the digital divide (71). Article authors also urged fellow researchers and other stakeholders to consider tailoring solutions and inquiries into this topic, as people are not a label or “part of a group” but in fact have their own unique needs and challenges regarding the digital divide.

First—studies demonstrated how we could expand our perception of groups who experience the digital divide. Several studies described people in terms of what type of technology they were excluded from or a characteristic that specifically divided them from using digital health technologies, instead of a demographic or cultural label. One study categorized people based upon behaviors and attitudes toward seeking health information online: worriers, a-digitals, skeptics, pragmatists, delegators and learners. These types were characterized by the combination of their beliefs about internet usefulness, learning attitudes, online enjoyment, trust in medical doctors, internet skills, internet interest, technology attitude, and self-efficacy. They even noted some overlap, e.g., that non-users were present in more than the a-digital group, further demonstrating that belonging to one of the six types is not mutually exclusive (35). Other studies based their analysis upon the resulting variation of digital literacy within their labeled study groups (34), health literacy, use, and motivation (36), ability and need to use the technology (37), and privacy concerns (38). They argue that these factors should be the basis of digital support needs and development, not demographics, and caution against generalization and related assumptions of digital competence or engagement. One study revealed that not only for those practicing Muslim faith, in which mammogram screenings were not allowed to be performed by men, non-Muslim and non-immigrant women felt similarly uncomfortable with this situation which resulted in automatic no-shows to the screening. Authors argued that it did not matter their socioeconomic, demographic backgrounds, but instead a level of discomfort and undesired service situation; “The point they wanted to get across was that having a Pakistani and/or Muslim background did not necessarily render them an “other” with respect to this, but rather united them with women with various other backgrounds” (58).

Second- while this review based the inclusion of articles on descriptions of why, how and who experienced the digital divide, there were also more optimistic results. Outcomes demonstrated that technology has a place and a time; included articles found that digital health interventions were successful in promoting digital inclusion amongst those whom they assumed were digitally divided. Authors credited participatory design and user engagement, and consideration of when certain technology was more appropriate, or preferred, than other situations. Even amongst groups who were commonly assumed to be digitally divided, studies demonstrated situations in which digital inclusion was possible. Some studies reportedly credited their use of end-user engagement and participatory design. The SlowMo app program was successful in engaging an arguably digitally divided group of people, i.e., women who are generally less confident in their use of computers, demonstrated higher rates of “current and future adherence to mobile app, usefulness and enjoyment” (34). The success of the Healthy Living for People with type 2 Diabetes (HeLP Diabetes) program in engaging those who were previously categorized as “digitally divided” based on ethnicity was credited to participatory design methodologies and the fact that the program was fully integrated into routine care. Recommendations to use the program by healthcare providers reinforced the support, motivation, trust and use of the program (36). Another study similarly suggested that support from warm experts, i.e., family and friends, could also overcome factors that classify someone as digitally excluded based on their vulnerable backgrounds, e.g., older groups and those who use mental health services. Situations in which these proxy agents could be most effective are when they could act as compensatory to a person's lack of skills, confidence and motivation to use online social and healthcare services. Therefore, incorporation of proxy agents in digital health interventions could include those otherwise considered digitally excluded (52).

## Discussion

To the best of the authors’ knowledge, this paper contributes the first CIS of literature describing the digital divide in health. It is through this process that we have identified that the knowledge provided by the CIS is nuanced, builds upon the knowledge provided by traditional review practices, and finally, aids in the further development of understanding the digital divide phenomenon in health.

Our findings indicate that the digital divide in health is a complex and dynamic phenomenon, consistent with many previous studies (83–85). We observe that this phenomenon frequently involves diverse patterns of use of digital health solutions. Many therapeutic interventions are effective only if used “correctly” or as intended, which demands continuous personal investment of time, energy, effort, and attention from users (86). However, this is not always an easy task, especially when digital health solutions fail to accommodate users’ personal circumstances (86, 87). For instance, many digital health solutions often overlook comorbidity and multimorbidity (87–89). Around 80% of people with diabetes have at least one co-existing chronic condition, yet most diabetes-oriented mobile applications do not take this into account (90). Additionally, we observe that the divide happens when digital health solutions emphasize short-term outcomes rather than a long-term strategy for maintaining patient engagement. One possible explanation is that these digital solutions are not sufficiently responsive to or reflective of patients including their caregivers’ beliefs, capacities, competing demands, and social support (25, 91, 92). Having realistic expectations for usage and allowing for flexibility and tailorable usage could ensure that users’ needs are met and that knowledge production is more accurate to real-world situations (87).

Consistent with many previous studies (83, 93), our results indicate that the digital divide frequently affects minority and disadvantaged groups. As evidence accumulates, it becomes clear that the most significant contributors to the digital divide are gender, age, education, quality of support, and privacy concerns (83, 94), which overlap with other forms of social inequality (95, 96). However, the digital divide is not solely determined by socioeconomic factors and biological characteristics. Traditionally advantaged groups, such as men, can also experience digital exclusion, while traditionally disadvantaged groups, like the elderly and female immigrants, can benefit significantly from digital health solutions. This suggests that, from the human aspect of care, the digital divide can fluctuate based on personal biography, such as motivation (93) and the tempo of healing (87). To address this, we should actively incorporate a community-based participatory approach when developing new digital health solutions, ensuring that human factors are not sacrificed.

### Knowledge provided by the CIS review

The CIS review builds upon the scoping review results. By acknowledging that most insights, themes, people, and technology appeared to be inextricably linked, the CIS method allowed us to explore the complexity of the digital divide phenomenon in health even further. In their literature review, Acharya states that, in reference to the connection between social and digital inequalities, “reiteration of an already explored understanding [levels] may not sufficiently contribute to the evolution of the digital divide's conceptual growth” (97). They argue that the common practice in which authors reiterate the definition of the digital divide from previous articles does not contribute to the re-conceptualization and development of our understanding of the concept (97).

A more comprehensive and accurate understanding of the digital divide is possible when its “levels” are instead seen as “areas”. Not only did this allow us to characterize what, how and who were inextricably linked, but it also points to an approach to knowledge generation that is more open to interdisciplinary perspectives. Levels portray static and hierarchical relationships between associated factors, e.g., access, skills and outcomes. Entangled areas are a more open concept that conceptually allows factors to change.

### Potential for knowledge translation: research to policy

Policy networks need knowledge to be translated into actionable information. Knowledge translation needs to know the goal (policy development) and have a foundation from which to build (relevant and actionable digital health information). Digital health in general is a multi-sectoral phenomenon—affecting and requiring participation of a variety of stakeholders (98, 99). This means that to effectively produce knowledge that is useful for all stakeholders, digital health research calls for interdisciplinary teams, including end-user involvement. However, this is a challenge due to differing priorities, vernaculars and methodologies (100). By providing a nuanced way of perceiving the digital divide, i.e., in terms of entangled and dynamic “areas” instead of “levels”, we argue that this CIS method can contribute to more and different research questions, and a more open and flexible communication between research disciplines and other stakeholders.

This knowledge foundation needs to be generated based on collaboration of stakeholders including the public or end-users, researchers, healthcare providers and policy makers and situated within the complex environment of digital health and the digital divide. The results of this CIS provide a new perspective of this knowledge foundation by: (1) including an interdisciplinary team of researchers (with the aim to include other stakeholders moving forward, as none of the presented paper's authors were implementation or policy researchers), (2) quality and diversity of digital health research methodologies, (3) inclusion of article authors’ opinions, based on their own experience in and interpretation of the field, and (4) diversity of factors including clinical, socio-political and personal.

As mentioned previously, the authors of this presented paper are not policy researchers and therefore we recognize that our interpretations and selection of data are based on other fields related to the digital divide in health. We suggest that by expanding the inclusion criteria and incorporating policy considerations it would be possible to “tweak” our original strategy, thereby including a more comprehensive CIS for use by policy networks and decision makers.

We need to ask new questions, look at the digital divide in health differently and critique our current assumptions to really understand this relatively new phenomenon. There is no shortage of theories that contribute to our understanding of who, what and how associated with the digital divide. However, a unified theory describing the divide, and the divide in health specifically, is still in development (101). In their book *Theorizing Digital Divides*, Jan van Dijk proposed four requirements for theory development: 1. basic principles, 2. fully defined and operational concepts, 3. empirical, verifiable, statements and use of 4. a heuristic approach to research (102). They argue that inter- and multi-disciplinary approaches are needed, given the range and complexity of societal realms affected by the digital divide in health.

### Strengths

As representatives of the research community, we hold a significant responsibility to explore, develop and challenge our current understanding. The inclusion of quantitative, qualitative and mixed-method studies provided a more comprehensive and thorough collection of digital divide knowledge. Because our reviewers were assigned to those articles that suited their expertise, we can be confident in an accurate extraction and interpretation of information. The CIS approach also allowed us the interpretive freedom to identify relationships between themes and risk factors (sub-themes) to provide more context, which can contribute to actionable recommendations. Most forms of literature review focus on compiling lists, which subsequently silo the information and make it more difficult to act. Understanding the digital divide is an example of needing to see the whole as more than a sum of its parts. The term “bridging the digital divide” is often used, which implies a connection of the two sides of a divide and, perhaps, that people exist on one side or another. For example, use of the reported characteristics of the digital divide in this paper demonstrates the complexity of which policy needs to address—not “targeted strikes” (forgive the military reference) based on lists of factors in separate sectors of society but multisector collaboration, including a representative portion of the population, adjusting current solutions and developing more inclusive solutions.

### Limitations

Inherent limitations of the CIS review itself are the potential for bias, similarly to qualitative analysis, as the results are interpreted by the reviewers and therefore subject to bias based on cultural, personal and professional experiences among others. CIS reviews are also not intended to cover a comprehensive set of representative literature and therefore run the risk of omitting potentially relevant literature (27). In this case, we limited our search to Europe, with the intention to align with the aims of the parent project, which was geographically focused within Europe (19).

We also acknowledge the limits of our approach to using the SDI method. While SDI analysis is typically applied to primary qualitative data, it was selected for this study to move beyond descriptive reporting and to foreground participant meaning as it is mediated through researchers' interpretations of the digital divide. Accordingly, published author interpretations served as our analytic “raw data.” We acknowledge that this introduces an additional layer of potential bias, as interpretations cannot be traced back to original participant accounts. However, alternative methods such as content analysis were deemed inappropriate because they emphasize summary rather than the synthesis and interpretive integration that define a Critical Interpretive Synthesis. To address this concern, we conducted a refutational analysis across studies and identified inductive codes through comparison of authors' interpretations. These were then mapped onto the current three-level theory of the digital divide. No single paper was weighted more heavily than another, and the resulting synthetic constructs reflect shared patterns across the literature.

We also did not include articles that described the testing of specific technology. This may have limited the number of articles describing the impact of the technology (3rd area). Thematic analysis and categorization of reasons for experiencing the digital divide were subject to some bias. However, this does support our recommendation that more interdisciplinary teams consider the evidence, discuss openly and find common ground to produce relevant, timely and actionable knowledge for policy. As this review was performed over a long period of time, we could not help but learn about and change our opinions and knowledge of the digital divide in health. While this is a part of the CIS method—and research for that matter—it did introduce some instability in our inclusion criteria. And while the authors of this review audited and, in some cases, changed the inclusion or exclusion of an article, this was done in such a thorough way that it is unlikely that many relevant articles were excluded based on our final inclusion criteria.

## Conclusion

The way in which we report study findings limits our ability to turn information into sustainable and successful action. True improvement requires a more comprehensive understanding of all moving parts of the situation—in this case, the digital divide. There exists a division between stakeholders—different factors, measures, theories, and vernaculars are used to describe the situation and are often reported in journals within their own fields and with different audiences. In other words, each of them is “preaching to the choir”. Translating all knowledge into practice, i.e., beneficial changes in policy and hopefully perceptions of all stakeholders, requires us to all get “the same page”, so-to-speak, to understand an almost “quantum” relationship between the factors of the digital divide in health. The diversity of our findings, and outcome of our interpretive synthesis, contribute to a new look at the digital divide—past static “levels” and toward a more dynamic and living ecosystem that crosses interdisciplinary divides.
