# Supplementary material for: Rethinking the digital divide in health: a critical interpretive synthesis of research literature
Source: Front Digit Health. 2026 Jan 15;7:1683565. doi: 10.3389/fdgth.2025.1683565 (PMC12852467; doi:10.3389/fdgth.2025.1683565)
Supplement: Supplementary file 2 [file Table2.docx]

# **S2 File.** Protocol for the review, analysis and synthesis of literature

## Critical interpretive synthesis approach

We aimed to follow the six characteristics of a CIS as outlined by Depraetere et al [1]:

1. A “compass” question that is able to evolve with the understanding of the literature as opposed to a static research question
2. A structured and broad literature search
3. Literature selection should be flexible and focus on all relevance of the literature, as opposed to only those that fit a strict set of inclusion criteria
4. The quality appraisal should aim to answer the “Appraisal prompts for informing judgements about quality of papers” described by Dixon-Woods et al [2]. Note: these are consistent with items of the Mixed Methods Appraisal Tool (MMAT) [3], described below in the *Quality Appraisal* section.
5. A reflexive data extraction and analysis process in which the authors acknowledge their bias in interpretation
6. A final synthesizing argument developed from synthetic constructs of data that aims to identify relationships between findings in the literature to form a “coherent theoretical framework”. [1]

As defined by Dixon-Woods et al, synthetic constructs are the conceptual format of the empirical evidence when all evidence is taken as a whole and weighted equally. Synthesizing arguments can be built from these synthetic constructs as well as categorized coded data (described below in the *Synthesis* section). These arguments rely on critical assessment of the evidence, its theoretical foundation and its quality, as presented in the reviewed literature. Dixon-Woods et al argue that this is more interpretive than standard quality appraisals of papers compared to study and reporting standards [2].

## Full search strategy

Two systematic database searches were performed in Web of Science, Medline (via Ovid), PsycInfo (via Ovid), and Sociological Abstract (via ProQuest) after consultation with a research librarian. One was performed on 22 February, 2023 and an updated search was performed on 23 January 2025.

Searches were limited to title, abstract, and keywords and followed the PICO search strategy:

[Population]: [list of European countries within the WHO European region]

AND [Intervention]: (digital OR technolog*) AND (tele* OR e-health OR ehealth OR m-health OR mhealth OR health OR healthcare OR health care OR medicine OR clinical OR care

AND [Comparison]: not applicable

AND [Outcomes]: Digital divide OR AND (divide OR gap OR inequalit* OR equalit* OR access* OR barrier* OR exclusion OR inclusion OR disparit* OR equit

AND [Limitations by publication date]: 2013-2023 (*an updated search was performed in 2025 for additional literature published between 2023-2025)

## Screening

**Round 1.** Screening for and exclusion based on titles and abstracts

Resulting articles were divided into 4 groups and two reviewers with research backgrounds were assigned to each group to review titles and abstracts independently. The online review platform, Rayyan, was used. This allowed for reviewers to be “blinded” to one-another’s decisions until review was complete and for reviewers to label each abstract based on reasons for uncertainty or exclusion (Table 1). Duplicates were flagged by Rayyan which made duplicate removal simple. Within the reviewer pairs, discrepancies between decisions to include or exclude were discussed and if necessary, the overall reviewer team was consulted until consensus was reached.

**Table 1**. Explanation of inclusion and exclusion reasons used during title/abstract review

| **Inclusion description** | **Exclusion reason** | **Label used during review*** |
| --- | --- | --- |
| Health related | Not related to human health, focused on employment, education or other non-healthcare related field | Not health |
| Full text available | Conference abstract without accompanying paper, abstracts of posters | No full text |
| Full text available in English, Norwegian, Swedish or Danish language | Full text unavailable in target languages | Wrong language |
| Study took place in Europe (as defined by the WHO) | Study took place outside of Europe (exclude even if European countries included) | Wrong location |
| Search words appear in the title/abstract | Search terms not found | Wrong focus (No search terms) |
| Participants are adults 18+ years old | Anyone under 18 years old was included as a participant or the focus of the study | Wrong population (Youth) |
| Primary research about those who experience the digital divide | Retrospective^α^ or document analysis | Wrong study design (Secondary research) |
| Primary research | Not primary research (Protocols, reviews, commentary, position papers) | Wrong publication type |
| Published in peer reviewed journal (with desired details for this review) | Conference paper |  |
| Target group described as experiencing the digital divide in health | Discussion of digital divide in health practitioner education, e.g. healthcare workers, students or researchers (may include if target group members are also participants) | Wrong population (Education) |
|  | Discussion of digital divide in healthcare practice, i.e. not about individuals’ or public groups’ (non)use of digital technology for their own health | Wrong population (healthcare practice) |
|  | Participants discussed, developed or evaluated only the positives of digital health technology, e.g. evaluation of an app without any mention of digital exclusion or divide, comment on the use or implementation of digital health or the focus was on development of a standard related to digital health use | Wrong outcome (description of technology, not use) Digital inclusion |
| Target group identified as those experiencing the digital divide before study start, prior to data collected, i.e. they are the only target group (participating or discussed) | The target group for the study includes others that are not identified a-priori as those who experience the digital divide (may be the same as Emergent) | Wrong population (Others included) |
| Not focused on COVID-19 related outcomes | Focused on COVID-19-related outcomes, e.g. impact of intervention etc. during COVID, impact of COVID restrictions, about vaccines or COVID (mis)information ^β^ | COVID |
| Participants are active participants | Development of research tool, e.g. survey validation | Wrong focus (target group members not the focus) |
| Target group described using the search terms | Participants are not described as digitally excluded or experiencing the digital divide (as defined by the authors) | Wrong population |

* Labels were also used when the reviewer was unsure whether to include or exclude the abstract. These were then collected and discussed amongst all reviewers.

^α^ If the authors of the paper reference their own work in which target group members participated or were discussed, such papers can be included, i.e. we aim for the authors to present primary research or their experience with primary research on the digital divide

^β^ If COVID is mentioned but not the focus of the paper or related to recruited individuals or reported measures, such papers can be included

**Round 2.** Full text review

Once a final list of abstracts and titles were agreed upon, full text articles were reviewed by three reviewers based on expertise and study type, i.e. MB with a mixed methods and quantitative background was assigned to review such articles using excel, and HLN and SAI reviewed qualitative articles using NVivo 13, based on reviewer preference. Full texts were excluded using the same criteria as abstracts and titles and labels were also applied. These labels provided reviewers with notes for discussion in the next phase (Round 3).

**Round 3.** Inclusion of previously excluded articles

Abstracts and articles that had been excluded based on 1) inclusion of participants outside of the target group, 2) lack of participation by target group were considered for inclusion.

The three reviewers considered inclusion based on presence of the following information in the article:

- Who did authors identify as those who experience the digital divide in health
- Why authors believed them to experience the digital divide, i.e. reasons or factors that contributed to the digital divide in health.

Primarily types of articles that were excluded and later included were commentaries, secondary research or data collection that did not include first-accounts from individuals but were reported by others about reasons for experiencing the digital divide amongst groups.

**Round 4.** Quality appraisal

Articles were assessed for quality in two rounds. First, articles were only accepted if they described receiving ethical approval or exemption and that participants signed an informed consent form. Articles that did not involve target group participants, e.g. commentary, were exempt from this requirement.

Second, formal article quality was assessed based on the Mixed Methods Appraisal Tool (MMAT) [3]. This approach is consistent with the “Appraisal prompts for informing judgements about quality of papers” described by Dixon-Woods et al for the appraisal of literature for a CIS review [2].

Two reviewers (MB and HLN) were again assigned to articles that described study types that aligned with their expertise. Five articles were exchanged between the reviewers to check findings. Consensus was reached based on agreement of exclusion reasons amongst the reviewers. A third reviewer (BW), who had expertise in quantitative studies, was consulted if agreement could not be reached.

Articles that were unable to answer quality appraisal questions or were unclear were labeled as “low quality” and excluded.

## Data extraction

MB and HLN performed data extraction. Information from all sections of the article were included to gain a more complete overview of current knowledge presented by the literature including the following: date of publication, country where study was performed, health condition, study type, intervention or type of technology described, target group recruited, reason for being considered target group, identified groups within recruited group, reasons reported for target group experiencing the digital divide.

Text from the articles were exported verbatim into an excel file to minimize bias. Reviewers noted terms used to describe groups and summarized reasons for experiencing the digital divide.

### Organizing extracted data

Based on emerging patterns and themes of data, reasons were grouped into Themes, i.e. levels of the digital divide, Primary sub-themes, i.e. categories of factors and Secondary sub-themes, i.e. factors. This is detailed below.

The first phase of organization involved noting specific groups, or subgroups, identified within the recruited target group and the unique reasons for experiencing the digital divide within each article (S4 File Table 1). Sub-groups were distinguished by the labels and descriptions provided by the authors in the results sections. Summaries for each reason for experiencing the digital divide were assigned with as many words as needed to denote specific influences yet as few words as possible to allow for feasible comparison and data synthesis.

The second phase involved specifying which technologies were described in each article. Upon extracting data, reviewers found it interesting that for participants to be eligible to participate, there were often technology-specific inclusion criteria which would likely impact the context of the results. Therefore, these associations were also extracted (S4 File Table 2).

The third phase of organization identifying which reasons fell under which “level” of the digital divide. The majority of articles described a three level digital divide framework based on Lavel 1: access, availability, Level 2: skills and motivation, Lebel 3: impact of the digital divide, i.e. lack of positive outcomes or experienced negative outcomes of using digital health technologies. We used this framework as the primary “Themes” under which we could categorize the reasons or factors of the digital divide. For articles that did not explicitly state which level they were describing, reviewers came to agreement based on the definitions of levels provided by other articles.

As data was being extracted, reviewers noticed that not all reasons fit solely within one level or another. Therefore, reviewers discussed the specific examples and agreed that a single factors or reasons can and should be listed under multiple levels and categories (S4 File Table 3).

### Analysis

Convergent qualitative synthesis was performed via thematic analysis in which all extracted data regardless of study type were transformed into qualitative data.

We were informed by the stepwise deductive induction (SDI) method. This abductive approach is generally applied to qualitative interviews, and we understand that while this method is meant for raw empirical data, we treated the article text as empirical data. The SDI method was appropriate for the CIS because the CIS was seen as a meta-ethnography of data transformed into qualitative data aiming to develop theory [4]. This work consisted of reading through the extracted data of the qualitative articles and coding emerging themes. The steps are:

1. Taking empirical data
2. Generating raw empirical data
3. Inductively processing data into codes
4. Taking the code-structured data and grouping codes or categories
5. Coding groups to develop a concept model
6. Discussing concepts to generate theory (which is not always possible) [4]

The fifth step can be seen as the development of synthetic constructs, and the sixth step can be seen as the development of the synthesizing argument in the CIS process.

The following is an example of this process taken for the presented article:


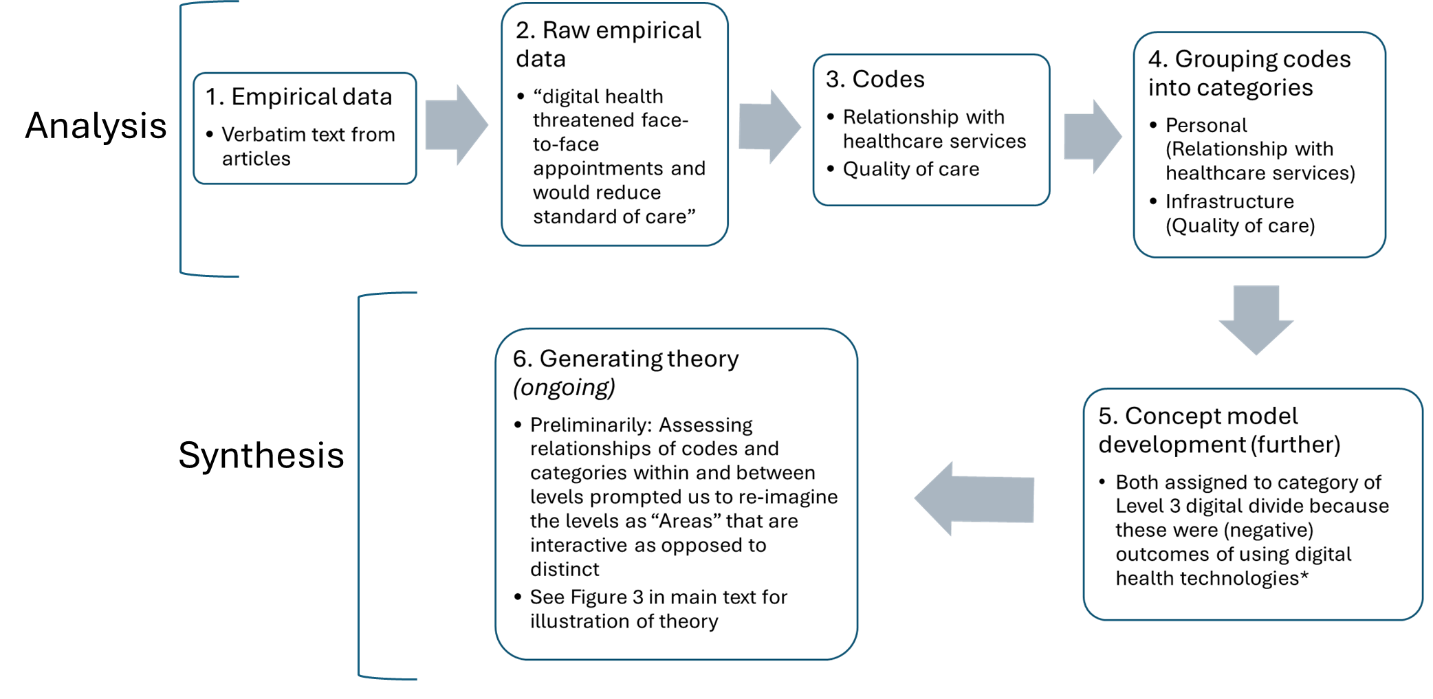


Fig. 1. Illustration of the stepwise deductive induction approach used to analyze and synthesize data

* Note: “Levels” of the digital divide were already well documented in the literature, so this was performed deductively

### Synthesis

This step encompasses the concept model development and generation of theory as described by the last two steps illustrated in Figure 1. More specifically, the concept model of the “3 levels of the digital divide” were questioned and reassessed for consistency with the empirical data.

An approach similar to a meta-ethnographic approach was taken to further develop the existing model of these 3 levels [5]. First, as described above, and similar to a Reciprocal translational analysis (RTA), we began by translating and integrating different types of information between and within articles into qualitative comparable data via thematic analysis while maintaining the integrity of the raw data by continuously checking themes with the authors’ intended meanings, i.e. raw data [5, 6]. While Dixon-Woods et al argue that this is not ideal for CIS because its aim is to translate rather than combine or converge findings, we chose this as simply a first step to be built upon by methods of synthesis in which we could converge findings [2].

Next, refutational analysis was performed to generate synthetic constructs. This is the process of critically synthesizing qualitative data from multiple sources and comparing arguments that authors present. It allows the researcher to vet findings against one another, i.e. explain reasons for contradictions or similarities [6]. Specifically, reviewers considered all extracted data equally and noticed relationships between topics, i.e. participants, technologies, factors, contexts etc. within each article text, followed by comparing findings across articles. Patterns emerged, for example 1) between individual factors within and across studies, 2) between participants and the technology, intervention or study types, i.e. what studies expected of participants, and 3) between participants’ personal situations or contexts, i.e. pre-existing experiences with technology, values, life priorities, responsibilities etc., and factors. The results section demonstrates how details, findings and arguments made in studies that differed in target group, technology, context, paradigm, study design and methodologies can all be speaking toward the same theory. A main example is the nuanced findings that reasons for experiencing the digital divide are cyclical, reciprocal, entangled and inextricably linked. The interpretations of these patterns of relationships in context led to generation of the synthesizing argument.

Lines-of-argument synthesis was used by taking the outcomes of the refutational analysis, further interpreting relationships and contextualizing them within a common model [7]. This was similarly described by Campbell et al. in their qualitative synthesis of experiences of diabetes [7]. We applied this approach by identifying the most predominant and complete constructs as well as granular arguments made in the articles about the reasons a group may experience the digital divide. We then addressed how and if the evidence could explain how each of these concepts or constructs could explain or elaborate on one another. The final synthesizing argument that explained all synthetic constructs was the evolution of the concept of “levels” of the digital divide into “areas” of the digital divide. This is further described in the results sections.

## References

1. Depraetere, J., et al., *The critical interpretive synthesis: an assessment of reporting practices.* International Journal of Social Research Methodology, 2021. **24**(6): p. 669-689.

2. Dixon-Woods, M., et al., *Conducting a critical interpretive synthesis of the literature on access to healthcare by vulnerable groups.* BMC medical research methodology, 2006. **6**: p. 1-13.

3. Hong, Q.N., et al., *Mixed methods appraisal tool (MMAT), version 2018.* Registration of copyright, 2018. **1148552**(10).

4. Lofthus, A.-M., et al., *“This is not a life anyone would want”—A qualitative study of norwegian ACT service users' experience with mental health treatment.* Issues in Mental Health Nursing, 2018. **39**(6): p. 519-526.

5. Noblit, G.W. and R.D. Hare, *Meta-ethnography: Synthesizing qualitative studies*. Vol. 11. 1988: sage.

6. Barnett-Page, E. and J. Thomas, *Methods for the synthesis of qualitative research: a critical review.* BMC medical research methodology, 2009. **9**: p. 1-11.

7. Campbell, R., et al., *Evaluating meta-ethnography: a synthesis of qualitative research on lay experiences of diabetes and diabetes care.* Social science & medicine, 2003. **56**(4): p. 671-684.
