# Supplementary material for: Rethinking the digital divide in health: a critical interpretive synthesis of research literature
Source: Front Digit Health. 2026 Jan 15;7:1683565. doi: 10.3389/fdgth.2025.1683565 (PMC12852467; doi:10.3389/fdgth.2025.1683565)
Supplement: Supplementary file 3 [file Table3.docx]

# **S3 File:** Extracted data

**Table 1. Specific groups identified and reasons for experiencing the digital divide**

| **[Recruited]** | **Specific groups Identified** | **Specific reasons** |
| --- | --- | --- |
| Patients with distressing paranoia from community mental health services [1] | Men | Discontinued use (including Technology does not support needs over time), one-time registration but lack of use, or less intended future use, Less engagement (disinterest or lack of desire), Lower perceived usefulness (after use) |
|  | Ethnic minorities, older people, Black people, women, People living in the inner-city (London) | Lack/limited tech knowledge, confidence, self-efficacy (literacy), or skills (lack training, skills are out of date/no longer relevant, or are too slow to keep pace) |
|  | Marginalized social, cultural, and demographic groups, ethnic minorities, older people, people living in inner-city London, Black people | Lack/limited access |
|  | Minority ethnic groups, men | Less engagement (disinterest or lack of desire) |
|  | Older people | Lack/limited tech knowledge, confidence, self-efficacy (literacy), or skills (lack training, skills are out of date/no longer relevant, or are too slow to keep pace) |
|  | Older people, People living in the inner-city (London), men | Less use or likelihood of use |
| interviewees (users and non-users) through their participation in the Oxford Internet Survey and in public places [2] | Delegators (subgroups of non-users and users), Pragmatists, Worriers, 90.6% of A-digitals (nonusers and users) | need improved accessibility to medication info (i.e. lab results, etc.), inaccessible digital platforms |
| Patients with T2D [3] | [No specific group] | Lack of equipment, Availability of technology, material deprivation (e.g., personal finances and living situation, Dislike text messages for health and lifestyle monitoring/advice |
|  | 75+ years old, People from mixed ethnicity group | Lack of internet connection |
|  | Older people, people from black and minority ethnic (BAME) backgrounds, people with less incomes, and people with less educational attainment | Not all have access to internet |
|  | Older people, people with disabilities, lower SES, low health literacy (general/health literacy is related to digital literacy) | low levels of access |
|  | People with low health literacy | consistently excluded from this area of research |
| Patients registered with the CIE patient-portal [4] | Those without education degree | Cost of accessing facilities needed to use eHealth |
| Respondents from Center data’s LISSPANEL, a representative population [5] | People with limited health/eHealth literacy (aka: vulnerable healthcare consumers) | Lack of or limited resources to support use |
| Representative of resident adult population (20+ years) from the Population Register Center [6] | Disabled internet non-users | transportation to screening facility |
|  | Users of online service | Lack of guidelines/information about eHealth programs and its content |
| Representative of resident adult population (20+ years) from the Population Register Center [7] | Older respondents (type of benefit not specified), Those in lower socioeconomic positions (type of benefit not specified), Less educated, poorer (fewer economic benefits), People who live alone (fewer social benefits), People with economic disadvantages, poor health, and a low level of participation (fewer economic and collaborative benefits) | difficulty accessing important info from website |
|  | People with economic disadvantages, poor health, and a low level of participation; Those with financial hardship/economic disadvantage, | Psychological barriers: awareness of the different types of technologies |
|  | Those who adapt to technology more slowly and limited in skill, Those in lower socioeconomic positions | knew nothing about how available digital health consultations work |
|  | Those who are less educated or unemployed, 22% of respondents, Those who are less connected or have fewer resources to use ICT | Lack of awareness of publicly financed digital healthcare or if their health center offered such a service |
|  | Those who are less socially connected/have fewer resources to use ICT | not aware of differences between different types of technology |
| Representative of resident adult population (20+ years) from the Population Register Center [6] [7] | Older respondents (perception of collaborative and economic benefits), Those with financial hardship/economic disadvantage (perception of economic benefits), 3% of respondents (perception of economic benefits), 4% of respondents (perception of social benefits), Those who are less connected or have fewer resources to use ICT, and associated negative attitude (perception of online benefits), Poorest and most marginalized groups, and those who are less educated or unemployed, and those living without a partner and low social participation, and poor health (perception of benefits, not specified) | Increased reliance on others as outcome |
|  | Older respondents, 6% of respondents (both groups perceive fewer health benefits) | Negative impact on self-perception (i.e. not having the ability/access to utilize digital tools has impacted the way they feel about themselves) |
|  | Internet non-users, Those who are older, have poor self-assessed health, more frequent hospitalization or disability | Negative mental wellbeing (note: from not being able to access online housing services because of lack of digital skills) |
|  | Older respondents (F1=1069.5, P<.001), Less educated (F2=39.4, P<.001), [No specific group], Those who are less educated or unemployed, 17% of respondents, those who are less connected or have fewer resources to use ICT | Negative experiences negatively affected their wellbeing |
|  | Older respondents (F1=266.2, P<.001), Less educated (F2=36.1, P<.001), Those who are older, have poor self-assessed health, more frequent hospitalization or disability, those who are less educated or unemployed | Negative emotional impact (i.e. feelings of loss of control and choice) (note: lack of belief and motivation (self-efficacy) in their ability to engage with digital tools leads to disempowerment) |
|  | Older respondents, large portion of population with higher unemployment and less annual income, vulnerable groups, 7% of respondents | Exclusion of feedback from those who are likely to have more severe problems regarding quality of service etc. |
| individuals who had engaged with institutions providing homeless services and family shelters [8] | 5.9% of homeless individuals | May not be ready to change the way they manage their condition |
|  | Homeless respondents (used at least one mHealth app less often and the internet for health reasons less often (11.2%) than the reference group (18.5%), Those who are homeless with serious chronic illness | Psychological barriers: knowledge of the different types of technologies |
|  | Those with educational barriers, Homeless populations who are at risk for limited health literacy | Lack of knowledge about what these services were, who provided them or how they worked |
|  | Vulnerable populations (those who are homeless, have educational barriers or physical barriers) | periods of time spent in inpatient care were also reported to be detrimental to participants’ awareness of advances in technological development |
|  | Vulnerable, e.g. Homeless individuals, those with physical barriers, and those with educational barriers, older people who are homeless | challenges accessing the online pension and healthcare systems due to knowledge requirements and digital stills |
| Case manager of patients with psychotic disorders and functional impairments from a specialized referral outpatient unit [9] | 43% of patients (reported by case managers) | cognitive difficulties associated with their mental health condition (e.g., memory difficulties) |
|  | 59% of patients (reported by case managers) | could not reach the kiosk because it was too high |
|  | 85% of patients, groups of patients with psychotic disorders and functional impairments, and older age and living in assisted living facilities, (also associated are: age, living situation, access to mobile phone, how they contact the unit, and how managers perceive patient’s ability to manage digital tech) | aging body as a barrier (note: impaired practical abilities, trembling fingers or impaired vision or hearing) |
| Pregnant women with legal residence from antenatal care centers [10] | exempt from e-Boks (majority non-Danish) | living situation |
|  | Lowest 25% of respondents, All ethnic minority respondents, non-Western immigrant respondents, Women exempt from e-Boks (majority non-Danish) | language |
|  | Western-born immigrants (vs. women of Danish origin), Majority non-Danish women exempt from e-Boks (vs. e-Boks users) | no access to internet (note: mainly because they do not wish to use it) |
| Home-dwelling persons with dementia, from memory clinics at local hospitals, municipal memory teams, and through advertisements in general media, and their informal caregivers [11] | 74% of people with dementia who had access to ATT | Cost of devices (note: as potential moderator of accessibility) |
|  | Caregivers and people with dementia | Want of technology that does not yet exist |
|  | Home-dwelling people with dementia, 74% of people with dementia who had access to ATT, People with dementia who lived with a spouse, or have older caregiver | affordability issues |
|  | Home-dwelling people with dementia, Caregivers and people with dementia | information was too complicated on website |
|  | Majority of people with dementia, People with dementia WITH access to ATT | Perceived financial barriers/unable to afford internet enabled devices/accompanying services/broadband |
|  | People with dementia who lacked ATT access | Financial cost, cost |
| Four generational cohorts from companies and retirement homes [12] | 65+ and part of baby boomer generation (age 59-77) | Poor quality internet, Internet too slow, Discrimination (note: concern about growing lack of consideration for those not interested in digital tools) |
|  | 65+ and part of Baby Boomer generation, Builder generation (born 1925-1945) | believed not all patients would benefit equally |
|  | Builder generation (born 1925-1945) | limited available information, a generational change was thought necessary before one would see an increase in the use of eHealth, Fear of discrimination and stigma (note: preventing them from being promoted at work) |
|  | College students 18-24 (Gen Y and Z) | Inequalities related to migrant/ethnic background as barrier to use |
| Persons with cancer from oncology or urology outpatient clinics [13] | Older people with cancer | Exclusions to use due to older age (i.e. challenges for older migrants navigating access to public sector) |
|  | Older people with cancer, Cancer patients who had completed treatment | More likely to be digital excluded due to social exclusion |
| Older adults and oldest aged 75-99 [14] | 75+ years old, Older females | social connectedness |
| Older Russian-speaking migrants who are active internet users, and third-sector representatives who assist migrants with digital service use [15] | [No specific group], Clients of third-sector personnel service (i.e. those with insufficient income) | Social exclusion due to long term health problems reinforces digital exclusion |
|  | Clients of third-sector personnel service (i.e. those with insufficient income, and have basic digital literacy but not at level needed for complex digital services), [No specific group] | Research does not accommodate older adults (i.e. not analyzed separately from other groups in research, interventions favor younger populations) |
|  | Older migrants who used internet but not digital services | material deprivation (e.g., personal finances and living situation |
|  | Russian-speaking older/50+ migrants, [No specific group] (Note: lack of e-identification needed for participation) | Technology represented ways of restricting freedom leading to digital alienation |
|  | Russian-speaking older/50+ migrants, [No specific group] (Note: lack of e-identification needed for participation), Older migrants who used internet but not digital services | Presumption of designers that everyone has the capacity and resources to use tech in the intended way |
|  | Russian-speaking older/50+ migrants, Living in metropolitan area, not being married/cohabitating, unemployed, pensioner or in “other” category, not having Finnish citizenship | material status (e.g. deprivation) |
|  | Russian-speaking older/50+ migrants, Older migrants who used internet but not digital services (Note: not having Finnish education, or outside labor market), Clients of third-sector personnel service (i.e. those with insufficient income) | Belief that the internet reinforces social isolation as it can become a barrier to interpersonal contact |
|  | Russian-speaking older/50+ migrants, Those with memory or deteriorating cognitive skills, [No specific group] | lack of financial resources |
|  | Those with memory or deteriorating cognitive skills, Clients of third-sector personnel service (i.e. those with insufficient income) | Few research interventions performed with older adults |
|  | [No specific group] (Note: prefer face to face with social care service officials, not specifically healthcare providers) | Concerns about sustainability of resources |
| Surgical patients from public day surgery units [16] | Non-users, | concern about the lack of social interaction and social skills of future generations |
|  | Self-selected older people (decline to participate in research), Self-selected less physically active people, Self-selected people less often to use a computer | Lack support (i.e. had to rely on others but if they are not available or if support does not have necessary skills) |
|  | Self-selected older people (decline to participate in research), Self-selected less physically active people, Self-selected people less often to use a computer, 60-69 years old, 75+ years old | limited resources to support use |
|  | Those with memory or deteriorating cognitive skills, [No specific group] | personal caregiver could be difficult to reach by phone |
| Persons with clinical epilepsy who were admitted for pre-surgical evaluation at the epilepsy monitoring unit [17] | 36.7% of Patients with uncontrolled severe epilepsy (Note: needed additional support/training), 13.3% of Patients with uncontrolled severe epilepsy (Note: needed constant supervision/help) | believed therapy should not be solely focused on a computer or on training people in specific skills |
|  | Patients with uncontrolled severe epilepsy (forgetting), 8 Patients with uncontrolled severe epilepsy (suboptimal use leading to non-captured seizures), 5 Patients with uncontrolled severe epilepsy (completely improperly used leading to 100% seizure activity not captured), [Participants with uncontrolled severe epilepsy with a higher BIPQ total score (higher disease burden)], [Participants with uncontrolled severe epilepsy higher “perceived disease timeline”/higher burden], [Participants with uncontrolled severe epilepsy higher “personal control”/higher burden] | Intrusiveness of technology and possible discomfort, concern about patients building rapport with the computer |
| Persons from closed and open prisons [18] | [Incarcerated people in Finland], [Incarcerated people in general] | perceived dangers of technological equipment |
|  | [Incarcerated people in Finland], Higher aged male and female prisoners of closed and open prisons in Finland (Note: also lower perceived control in use of digital services), Those with more convictions (Note: also lower perceived control in use of digital services) | believed apps requesting information was intrusive |
|  | Incarcerated people in Finland | concerns about inaccuracy/meaninglessness/effectiveness of data gathered by tech, felt that a smartphone application for monitoring their eating habits would take control of their lives, lack human contact and impersonality of computer use (i.e. inability to context someone for advice/clarification, lack of face-to-face) |
| People with vulnerabilities in mental health from organizations providing community-based mental health rehabilitation, support, and sheltered employment services [19] | 37.2% of adult mental health service users who do not use digital services (Note: can solve problems themselves) | Would need additional support (note: in order to accept technology) |
|  | 37.2% of adult mental health service users who do not use digital services (Note: can solve problems themselves), 9.1% of adult mental health service users who do not use digital services (Note: because did not receive needed help from anyone) | Required assistance from Finnish national to file paperwork (note: because lacked sufficient knowledge about the social benefit and perhaps the necessary Finnish language skills) |
|  | Adult mental health service users in general | Needed instruction and/or help |
| Adults from patient organizations, online from hospitals and health clinics in multiple countries [20] | [no specific group/general population] (Note: general population found to have limited access to certain digital technologies | Required help from others (note: no participant had learned to use digital services themselves, but instead with help of people close to them), Limited access to certain digital technologies |
|  | 24% migrants who do not wish to access PHRs (71% of whom reported difficulty living on present income) | need for training or support |
| Random sample representative of the population ID’d via population registry [21] | [No specific group] | Age |
|  | [No specific group] | Education, income etc. |
|  | Male respondents with poor mental health, older individuals with mental health disorders, Those with mental health disorders, | Less engagement (disinterest or lack of desire) |
|  | Older individuals with mental health disorders | Lack/limited access to the technology, Fewer non-health outcomes |
|  | Older individuals with mental health disorders, older adults, socially marginalized groups | Lack/limited tech knowledge, confidence, self-efficacy (literacy), or skills (lack training, skills are out of date/no longer relevant, or are too slow to keep pace) |
|  | Socially marginalized groups | Inability to use digital technology themselves/ needed assistance |
|  | Those with mental health disorders | Social exclusion (loneliness, social loss, lack employment, stigma, lack of citizenship etc. contributes to inability to use), or less social support in general, Limited/lack knowledge of healthcare system or services |
|  | Those with mental health disorders, those with severe mental health problems, Vulnerable groups, older adults, Women experiencing poverty, Male respondents with poor mental health | Lack of trust (including data safety/security/confidentiality, or unwilling to share personal info online) |
|  | Those with mental health disorders, Those with severe mental health problems, Vulnerable groups, older individuals with mental health disorders, older adults, Those with less education, University students with depression | Negative or less positive beliefs, attitudes and/or emotions toward use (including low perceived usefulness, anxiety, discomfort, fear of misunderstanding) |
|  | Those with mental health disorders, those with severe mental health problems, Vulnerable groups, Women experiencing poverty, Male respondents with poor mental health, University students with depression, Marginalized young people | Prefer traditional forms of communication (e.g. face-to-face, or offline support) |
|  | Those with mental health disorders, Vulnerable groups | Discontinued use (including Technology does not support needs over time), one-time registration but lack of use, or less intended future use |
|  | Those with mental health disorders, Vulnerable groups, University students with depression (difficulty using services when depressed) | Less engagement (inability or difficulty) |
|  | Vulnerable groups (deficient financial resources causes cynicism toward digital services), [No specific group], Older individuals with mental health disorders (financial resources) | Fewer offline resources or material deprivation |
| Expert developers and distributors of digital therapeutics from digital health databases and press searches [22] | [No specific group/general population] | Improper use (sub-optimal activation/adherence or forgetting to perform step properly for use) |
|  | Patients who have illnesses requiring high patient awareness (Note: the expense is because financially unviable to inform each patient individually since specific illness), [No specific group/general population] | Actions of healthcare providers, insurance companies or other health authorities |
| Non-emergency surgery patients from outpatient department [23] | 22.6% of surgical patient participants (Note: would not trust technology to make health decisions), Young adult surgical patient participants | lacking confidence and ability needed to try new things |
|  | 26.8% of surgical patient participants (did not know if it was useful), 49.6% of surgical patient participants (did not believe it was useful) | did not possess necessary skills for electronic web services |
|  | 26.8% of surgical patient participants (Note: did not know if it was useful), 49.6% of surgical patient participants (Note: did not believe it was useful) | Lacked knowledge of benefits |
|  | 38% of surgical patient participants, Older surgical patient participants (disinterested in video consultations) | Unfamiliarity with computers or tech in general |
|  | 55.4% of surgical patient participants | Lower digital literacy |
|  | Older surgical patient participants | Insecurity with use of digital tools (e.g. do not trust their own knowledge and capabilities) |
|  | Older surgical patient participants (Note: less frequently searched for health info online) | Low confidence in the electronic medical record (note: leads to a greater mistrust of digital tools) |
|  | Older surgical patient participants (Note: less ready to use ICT for health) | Insecurity with learning new things (e.g. digital tools) |
|  | Older surgical patient participants (Note: less likely to have learned to use computer), surgical patient participants who didn’t understand ICT | insufficient knowledge of system (note: influences the experience of adoption) |
|  | Older surgical patient participants, 39% of Older surgical patient participants and Surgical patient participants with less education (Note: did not know which operating system their phones used) | stress of not being able to keep up with knowledge needed to handle digital tools |
|  | surgical patient participants who didn’t understand ICT | Grew up without internet, Not being able to keep pace with knowledge |
|  | Surgical patients (Note: percentage of lack of access varied depending on type of technology), older surgical patients | Lack of computer literacy |
|  | Surgical patients (Note: percentage of those who did not own technology depending on type of technology | lack experience and knowledge of digital tools |
| members of a Diabetes organization with T1 and/or T2D [24] | 75+ years old, Less than high school education, Very low income groups (Note: less use of eHealth), People with T1D of middle-and high-income groups (Note: less use of video services), Men with T1D and T2D (Note: less use of social media), Men with T2D (Note: less use of search engines), Older groups with T1D (Note: less use of apps and search engines) | (Previous literature) lack of skills or knowledge (note: contributed to only 36% reported having ever used the internet) |
| Immigrant women with a Pakistani family background, and persons with experience and/or positions of relevance [25] | (Canada related lit) Pakistani and other South Asian countries | not having adequate skills to applying for benefits (note: needed to use email and how much longer this takes when he has to ask his employer to assist with this), Late adaptors (note: contributes to computer anxiety and poor understanding of usefulness) |
|  | (Denmark related lit) Pakistani immigrants | lack of digital skills needed to access online housing services (note: negative impact on mental wellbeing) |
|  | (Other literature) People from Pakistani and other South Asian countries living in Canada | knowledge issues, lack of knowledge or skills |
|  | 59% of Pakistani immigrant women | Lack of previous experience |
|  | Older Pakistani immigrant women in Norway | Confusion over how to use web-based services, concern and fear of using tablets and technology (note: due to lack of knowledge or low confidence or perceived dangers of technological equipment), not having adequate level of digital skills and appropriate digital equipment (note: impacts on her employment options) |
|  | Pakistani immigrant women in Norway | A perceived lack of knowledge, lack of knowledge and confidence (i.e. concern and fear of using tablets and technology in general due to lack of knowledge or low confidence or perceived dangers of technological equipment) |
|  | South Asian immigrant women in Norway | Lack opportunity to learn how to use digital tools (i.e. lack training) |
|  | Younger South Asian immigrant women in Norway | Lack of technology experience |
| Elderly patients (65–80 years) with a chronic disease from primary health care centers [26] | Elderly patients with a chronic disease | being inexperienced in using the internet (note: may be intermediate mechanisms leading to non-adherence), felt they lacked the necessary digital competence to use technology effectively, Challenges with trying new technologies, Psychological barriers: confidence , did not feel that they had the ability to distinguish between legitimate websites, lacked understanding of How to navigate security and verification processes, lacked technical literacy , technically illiterate, Trouble knowing what button to press/how to navigate website, Low acceptance (note: influenced by low digital literacy), Difficulty completing task of answering questions via kiosk |
|  | Elderly patients with a chronic disease and some experience in eHealth | Insufficient introduction use of smartphones, Low engagement or decision to use technology (note: moderated by severity of symptoms) |
|  | Persons with experience with electronic medical record | Low digital competence, lack of experience and skills (note: was holding her back from trying new technologies) |
| Persons currently experiencing clinically significant symptoms of major depressive disorder in multiple countries [27] | Participants in UK and Italy with depression | Cognition (i.e. problems with memory, reading, expression), Symptoms of depression (i.e. intermittent poor insight, memory), Lower incidence of breast cancer (note: lack of incentive) |
|  | Participants in UK, Spain and Italy who do not own mobile technologies | health-related barriers |
|  | Participants with depression in Italy | Physical and health issues influenced technology use (i.e. fitness and mobility issues particularly in relation to technology such as activity monitors), additional risk of increased anxiety between regular visits at PHCC |
|  | Participants with depression in Spain | effect that medication can have on participants and their ability to partake and complete these programs |
|  | Participants with depression in UK | Cognitive decline (note: despite being open to use), Fear that use will cause health anxiety, Invited but did not attend screening, Potential negative impact on relationship with physician |
|  | Participants with depression in UK, Spain and Italy | lack knowledge of breast cancer, Competing life stressors/priorities (note: left little room for screening despite knowledge of disease and screening program), Forgetfulness, (Previous literature) cognitive difficulties (note: contributed to only 36% reported having ever used the internet ), symptoms of depression (i.e. lack of motivation or interest to carry out activities (anhedonia) limit pursuit of rewards), Difficulty to adhere to treatment during times of crisis, disability, compromised in their ability to achieve investment in interaction with tech, Difficult to interact with touchscreen, insecurity and fear of not being able to manage access to healthcare in emergency situations, lack of awareness and understanding of different technologies and how these could benefit them (note: despite using technology for specific tasks), Concerns about technology limiting relationship with healthcare provider, various degrees of skepticism toward digital health consultations (i.e. these types of meetings were too impersonal) |
|  | People with depression in general | poor memory and sensory impairments, Symptoms of mental health difficulties (i.e. specifically psychosis and relapses and hallucinations preventing them from being able to use internet-enabled technology and forgetting how to use the technology/memory difficulties), Anxiety about one's health (note: associated with cost) |
| Persons 50+ years who suffer from chronic pain (some recruited from a previous eHealth research study) [28] | Middle-aged and older adults with chronic pain | Prefer letters in the mail over electronic messages, Prefer personal contact, Face-to-face support preferred (note: to avoid misunderstandings), preference for offline interaction , Missing the human touch/valued human interaction/preference for offline options , valued human interaction, Reluctant to be touched by male providers, Satisfied with the current use of primary healthcare system, not felt the need for alternative to primary healthcare system, satisfied with traditional health information sources (i.e. written or oral from trusted sources), had not engaged in the development of digital consultations, ethnicity, gender, Language as barrier to use (i.e. hard- ships with understanding the online forms in the Finnish language), living situation, keeping up with the momentum required by eHealth programs and the failure to establish and adhere to a routine, Reasons for disengagement included difficulty changing lifestyles (i.e. conservativism, previous non-digital routines and habits), Reasons for disengagement included motivation (i.e. laziness) , Reasons for disengagement included negative emotions (i.e. fear e.g. when discussing online public services of Kela and employment office due to language barriers, computerization and hardships experienced in the past, distrust, anxiety/stress despite being an active user of multiple services), Discontinued use (i.e. difficulty dealing with requirements of tech), Discontinued use (i.e. difficulty dealing with requirements of tech) |
|  | Older adults | Prefer face-to-face contact with healthcare providers, Face-to-face support preferred during periods of crisis, age, old age prevents them from use |
|  | Rural parts of the country | Feel more trust/safety with in-person over visual consultations, Privacy (i.e. of filling out personal information in public setting, including healthcare staff who were situated directly next to the kiosk), privacy and safety concerns (e.g. online fraud, or knew someone who experienced negative consequences of use), anxiety about recording of data by tech (e.g. virtual assistant), frustrations with computer breakdowns and their trouble navigating on the internet, Lack of support to access health and preventive care (note: “digital inverse care law” - those who most need the benefits that come with digital health tools also being the least likely able to access it), Lower rates of diagnosed breast cancer or more advanced due to low screening, Digital exclusion could lead to diagnostic overshadowing (i.e. when new symptoms/physical health issues are wrongly attributed to the persons mental health condition), interaction with touchscreen buttons would cause pain |
| Elderly Russian-speaking migrants who were not digitally proficient from workshops that included digital services in their curriculum [29] | Elderly Russian-speaking migrants | Lack of trust, privacy |
|  | One participant elderly Russian-speaking migrant | nervousness and lack of trust that can accompany allowing others to use or share this information (data security) |
| Patients from a large secondary mental health provider who were digitally excluded [30] | (Previous literature) community mental health service users | mistrust of the internet (note: due to negative previous experiences) |
|  | (Previous literature) outpatients diagnosed with schizophrenia | Lack of will to engage, do not wish to access the internet |
|  | Mental health service users | lack interest in digital solutions, Lack interest in technology (note: may affect behavior/habits in use of web-based health info), no interest |
| Community-dwelling healthy older adults [31] | Community-dwelling healthy older adults | Limited experience using PHCC's website to contact primary care doc, Low engagement |
| People with severe mental illness from community organizations who are digitally excluded [32] | People with severe mental illness | Percentage of those who used tablets to go online was low, Observed participation was low, low levels engagement, rarely used the internet, Psychological barriers: motivation , Felt lack of energy and motivation, Negative experiences (i.e. services demonstrating a lack of awareness around digital exclusion, previous attempts to engage with digital tools which had not been successful (note: negatively affected their wellbeing and their motivation to engage with digital tools again in the future), negative experiences (note: despite using technology for specific tasks), negative past experiences (note: led to fear of "getting it wrong"), Skeptical attitude about benefit of tech (note: as moderator of engagement) |
|  | People with severe mental illness who are non-users of social media | lack of instructions and guidance (i.e. if there are any instructions, they are too technical, when asking for assistance, other people quickly completed the job for them instead of guiding them) |
|  | Second Participant with serious mental illness | had some kind of smartphone but only used few functions |
|  | Third participant with severe mental illness | Lack motivation |
| Staff, patients people with long-term conditions and carers in the implementation and evaluation component of study about digital patient feedback system [33] | (Previous literature) People with disability | poor understanding or lower perception of usefulness |
|  | Older people | fear of control (Big Brother is watching me), belief that staff may distrust computers |
|  | Patients in general who use the health care services | Non-use, Mastering tech is intimidating , felt that there are too many pieces of technology, Felt nervous about possibly deleting information (note: despite familiarity with computers), Perception of time allotted , Believe many elderly are afraid of this type of tech, uncertainty and concern about answering questions about cookies , Skeptical about searching on internet and about information found, Difficulty solving computer problems (note: despite experience) may reduce someone's ability to complete eHealth program course, Perceived disadvantages of using tablets (i.e. weight/heavy), potential danger of the computer becoming a “gimmick” (note: which then dilutes the relationship with a therapist), wary as to whether other clinicians would welcome computers in the context of therapist-delivered interventions |
|  | Patients with hand impairments | (Previous literature) higher rates of internet non-use, none used computers for substantial therapeutic functions in routine practice (note: due to beliefs of potential confidentiality threats for people with ID) |
|  | Patients with long term health problems who use health care services | (Previous literature) only 36% reported having ever used the internet (primary 1st level barriers: financial costs, and access), Lack of knowledge and skills |
|  | People who were not familiar with computers | confusion over various technologies and terminology (note: despite using technology for specific tasks), Believe tech as sub-standard option |
|  | People with mental health problems | Perception of effort, Technology was intimidating, Risk that it may dilute the relationship with a therapist |
|  | people with physical limitations in general | Concerned about accidental deletions, Potential low digital literacy of healthcare professionals |
|  | Those who were concerned about accidental deletions | Afraid will not to be able to keep up with its use as grow older |
|  | Those without previous experience of use | distrust towards computers as a sub-standard option (note: driven by financial motives and not by therapeutic reasons, often influenced by their own lack of knowledge and skills in this area) |
|  | wheelchair users | Choice to be non-user |
| Service users with intellectual disability (who had previously taken part in a computerized therapy skills training session) [34] | (Previous literature) Clinicians | fear that technology may replace human contact and put more pressure on healthcare personnel/ lack of time for physicians, Belief that People with ID do not have capacity or skills for computers , Fear and concern for the concept of digital consultations (i.e. either about themselves or older people having difficulties using various digital technologies), Cost of technology, Financial motives, Potential for perceived cost to outweigh perceived utility , |
|  | Clinical psychologists | Potential that physicians may miss something that would be necessary to derive diagnosis, lack of continuity among care- givers, Skepticism based on financial concerns (i.e. private care providers were exploiting the reimbursement system for these consultations and wished to take a firm stance away from this), Fear/insecurity of reduced accessibility to healthcare for the elderly (note: because younger patients overuse the system), risk of increased cost, cost (high price of tablets and other technological equipment , |
|  | people with intellectual disability (ID) | concerned that the elderly might get left behind and excluded in the digitization of healthcare, Potential burden on healthcare providers' resources , |
|  | People with intellectual disability and Clinical psychologists | Potential negative impact on care (i.e. may not be available to help process information, and signs of health deterioration may not be acted upon even if discovered), logistical issues with the provision of, and responsibility for, the necessary hardware and software and for staff to have the necessary time and skills, Importance of balancing utility and cost |
| Elderly persons (65+) living in urban countryside (rural areas) from senior citizen meetings (who had not used digital health consultations before) [35] | Elderly | Digital modality did not suit them (note: despite wanting to give feedback), Multiple choice answers often too similar |
|  | Elderly persons (65+) living in Swedish, urban countryside (rural areas) who had not used digital health consultations before | eHealth program timeline insufficient for successful completion, Inability to give feedback about program to program director about efficacy, Digital modality did not suit them (note: despite wanting to give feedback), Digital modality did not suit them (note: despite wanting to give feedback), Program content often neglect aspects of condition that impact their lives (i.e. depression/ Failure to acknowledge that depression can be part of CP, family and friends misunderstanding the seriousness of their condition), Program did not offer information to help them cope daily, Poor features of PHCC's website (i.e. lack of functions and unmet expectations of e-health) |
|  | Few respondents | believed digital consultations were a generational matter (i.e. not designed for the elderly population), Difficulty navigating eHealth programs, uploading info, opening links to program materials, viewing videos on small monitors, starting/pausing/stopping videos and changing text to a larger font for easier reading, problems with passwords, setting up interactive devices |
|  | Most respondents | Appropriateness of tech (i.e. doubt about use of computers in therapy without personalized approach for people who have complex emotional and social needs) |
|  | One participant | frustration that my smartphone doesn’t understand me (note: reverted to handwritten notes and notebook calendars), Inadequate information to assist older adults self-manage |
|  | people in rural areas | eHealth programs/content lack acknowledgement that counseling in primary care can be a benefit |
|  | Some respondents | Uncertainty about relevance to participants and their condition, perceptions that technology is for younger people or too difficult to learn at an older age, Lack of information about new treatment options in content, Inability to receive tailored feedback, Content/ instructions difficult to follow (e.g. repetition and a lack of flow and logic to the program content) |
| Patients with colorectal cancer after hospital discharge, their informal caregivers, and HCPs [36] | Patients with colorectal cancer after hospital discharge and informal caregivers | negative perception of self and disempowerment |
|  | Patients with colorectal cancer after hospital discharge who claim that they are the last generation not to use ICT | Adoption and use difficult due to complexity of system |
|  | Some patients with colorectal cancer after hospital discharge | Image quality of video consultations as big concern, concerns about increasing dependency on tech, technology felt as though meant for younger population, Lack of empathy provided by using tech (i.e. left some feeling like a statistic), Negative emotions (i.e. feelings of discomfort due to shared public space of waiting room where kiosk was set up), over-reliance or dependence on technology which could lead to physical inactivity and laziness |
|  | Some patients with colorectal cancer after hospital discharge and informal caregivers | Negative emotions (i.e. stress and anxiety), computer anxiety (i.e. fear of failure or lack of confidence in using new technology), potential negative impacts of tech (e.g. addiction) |
| Adults from database of households currently participating in a project [37] | (Other reported results on this study) 101 (21%) respondents | Physical setting of technology (note: led to feelings of discomfort because healthcare staff who were situated directly next to the kiosk), Knowledge demands on elderly are too high, Expectations of participants about level of engagement with touchscreen of kiosk was unrealistic, Requirement to apply certain amount of pressure to buttons on touchscreen was barrier to use (i.e. physical limitations/requirements of tech) (note: different types of digital screens demand variable embodied orientations involving an ‘immersive investment’ of the senses, including the hand), lack of belief and motivation (self-efficacy) in their ability to engage with digital tools |
|  | (Other reported results on this study) social housing community involved in the Smart line project | Privacy/security concerns (note: also seen as personal cost or risk) |
|  | Many rural social housing residents | Expectations of participants about level of engagement with touchscreen of kiosk was unrealistic |
|  | Most rural social housing residents | increase the awareness of living with a chronic disease (note: might indirectly intensify the experience of distress, which may lead to rejection of digital interventions) |
|  | One rural social housing resident | Requirement to fix technical malfunctions, Interface usability issues (note: despite being open to use) |
|  | Rural social housing residents with T2DM | concerned that users need to be technologically knowledgeable to use these kinds of healthcare services, Concerns about spending time and money (i.e. cost of engagement) |
|  | Several rural social housing residents | Practical challenges with use (i.e. losing opportunities to log data due to appearance of notifications at inconvenient timings, the need for charging, and the loss of connection Disappointment in healthcare's poor use of IT systems (i.e. lack of IT competence in organizations, poor communication between organizations' IT systems) |
|  | Some rural social housing residents | beliefs of potential confidentiality threats for people with ID, Expectations of participants about level of engagement with touchscreen of kiosk was unrealistic, touchscreen of kiosk posed issues (note: knowledge of using a phone was not transferable to this technology), Lack of access to health services or alter type of health input available to them (note: which has negative emotional impact on those with mental health challenges), Difficulty physically interacting with touchscreen could lead to people aborting their attempts, or not returning after their consultation |
| Persons with T2D diabetes who are disadvantaged sub-segment of the community, requiring utmost care and consideration, including presence of comorbidity and risky lifestyle behaviors, have poor diabetes management, have low education and health literacy, from outpatient clinic [38] | Most informants of vulnerable people with diabetes | Non-use reinforces or increases social disconnectedness (e.g. more difficult to hear about community events), Feelings of abandonment after eHealth program is over (e.g. lack of support) |
|  | older informants of vulnerable people with diabetes | Poor life expectancy (i.e. people with SMI dying on average fifteen to twenty years earlier than the general population) (note: “digital inverse care law” - those who most need the benefits that come with digital health tools also being the least likely able to access it), Negatively impact employment options (note: because does not have adequate level of digital skills and appropriate digital equipment), poverty , social isolation, feeling of disconnect with wider society |
|  | vulnerable people with diabetes | Frustration of carrying out learned elements of program on their own/without support |
|  | Vulnerable people with diabetes who used personal caregivers | negatively impacts financial stress (note: because negatively impact employment options because does not have adequate level of digital skills and appropriate digital equipment), Perceived stigma (i.e. second layer of stigma of digital exclusion in addition to mental health stigma, reinforced by others assuming a certain level of digital inclusion, not using digital tools can influence how they perceive themselves to be viewed by others and their place in society) |
| Adults (70+ years old) with chronic kidney disease who lived in areas of deprivation [39] | Some participants | Lack of engagement with digital technology, lacked necessary skills, resources and support, lack of awareness of and access to community/charity-run digital support |
|  | Participants who were less digitally abled, those who had done jobs that did not involve using computer, Older retired persons | Lack of opportunity in the workplace to become digitally proficient, or entered retirement before workplace began using digital systems (missed opportunity to learn) |
|  | Most participants who never used digital technology for their health | Lack of access to digital technology and infrastructure, (some) did not own smartphone device or computer and had poor mobile phone reception or lack of good access to internet at home |
|  | Those living in socially deprived areas | financial instability |
|  | Those with little or no access to digital technology | Lack of support to use technology by healthcare providers or family, those who could teach them (family and friends) did not live nearby |
|  | Those with support for digital technology by family or friends | Lacked opportunity to learn new digital skills (supporters did it for them) |
|  | Many participants | Disinterest, lack of motivation, believed that technology was not relevant for them, lacked capacity to learn, age, expected to be unsuccessful in attempts to learn, preferred their healthcare providers to take responsibility for health monitoring, burden of monitoring their health had negative impact on their mental health, technology does not meet their needs, complicated user interfaces and do not accommodate complex physical and cognitive needs, presented content was too generic, setting up technology is not user friendly, frustration and confusion contribute to low engagement, tech often not fit for purpose, difficulty accessing and understanding information, receiving too many notifications, digital health threatened face-to-face appointments and would reduce standard of care, preference for in-person appointments, digital health perceived as insufficient for health assessment, cold and impersonal, concerns about profit driven vendors |
|  | Older individuals living in areas of higher social deprivation, many other participants | anxiety about using technology correctly, making mistakes, previous negative experiences, feeling helplessness and infantilization |
|  | Participants with high disease burden (e.g. older age, chronic illness and competing physical and cognitive morbidities) | Poor finger dexterity, general mobility issues, poor eyesight made it difficult to use tech, poor memory made it burdensome for them to engage with self-monitoring tech, e.g. remembering login details |
| People with an without intellectual, visual, calculation, language, impairments [40] | Participants with impairments (compared to those without) | Less use and more difficulty using eHealth, difficulty booking appointments online, avoided using eHealth, underrepresented in eHealth research, accessibility standards are too narrow, technologies lack cognitive accessibility |
|  | Participants with communication, language, calculation and intellectual impairments | Least use and most difficulty with eHealth |
|  | Participants with communication, language, calculation, intellectual impairments, and visual impairments | Less use of digital identification, more frequently avoiding booking appointments online |
|  | rare populations | hard to reach by conventional methods in population studies |
| People receiving long-term dermatology follow-up [41] | Digitally excluded individuals, 10 million UK residents with unequal access and capacity to use technologies required to participate in society | Low technology use, poor telephone facilities, difficulty with photo sharing (i.e. use of remote dermatology tech), |
|  | Groups 3 and 7 | Lower health technology readiness, vulnerable to digital exclusion |
| People (18+ years old) who had and had not previously used NHS 111 online [42] | People with no formal qualifications, Those without LTHC (compared to those with) | Less likely to use NHS 111 online |
|  | Non-users (vs users) of NHS 111 online, baby boomers and older adults seeking information online, Those with long-term health condition | Lower eHealth literacy |
| Post-discharge stroke survivors with aphasia and milder cognitive and physical disabilities [43] | Participants (number not specified) | Needed time and training to understand tool, difficulties reading/understanding amount of text, difficulty understanding concepts such as SMART goals, color coding was not clear, eHealth service design does not include target group |
|  | Group 1 participants | Challenges using tool (did not accomplish tasks successfully), |
|  | Those with cognitive impairments | lack of access, lack of availability, assistive tech is often too complex |
| Frail older adults who had completed most or all of the +AGIL Barcelona, “A community programme of integrated care for frail older adults”, and providers [44] | Frail older adults | Low use of online digital technologies, low predisposition/strong opposition to use tech due to lack of skills, problems finding balance between personal activities and attendance to intervention programme, laziness, lack of motivation to exercise alone at home, incentive to be physically active reduced after study ended |
|  | Some frail older adult participants | Current technologies used were too basic to allow for variety of use possibilities |
|  | Frail older adults (reported by professionals) | too complex, have low learning capacity, unaware of their own digital divide, do not know usefulness, prefer face-to-face appointments, misperception or lack of awareness about the purpose of the program |
|  | Frail older adults and professionals | Self-perceived health status, i.e. physical limitations or comorbidities such as pain and self-perceived frailty), expectations, COVID restrictions caused lack of mobility, difficulty continuing the program at home (instructions difficult to continue) |
| Young adults with a diagnosis of autism and/or ADHD who use support services, and their support workers [45] | some clients (neurodivergent young service users’ (ages 18 to 29 years) | need face-to-face contact, changes in the relationship and communication may lead to poorer quality care, challenges concerning the feasibility of digital interventions were highlighted, including the issue of maintaining data privacy and technical issues, questions about effectiveness in enabling young adults to learn and retain skills |
|  | A few support workers | They had no interest in using remote support, |
|  | Many support workers | a preference for in-person meetings, and that remote support does not suit all young adults or support workers, expectations to always be available can be stressful and make their work more unpredictable (which may force the support workers to make spur-of-the-moment decisions about time allocation, which may lead to an unfair distribution of support.), meeting another person and having continuous contact is at the core of the support service, including building a relationship, adapting one’s behavior, and setting boundaries, managing sensory sensitivities (e.g., contact with water or dirt) could be difficult to do remotely. |
|  | Most service users | negative attitudes towards remote support, and some stated that they would rather cease the support service than only have remote contact, software problems, lack of network connection, and added costs, did not want to bother their support worker and may refrain from initiating contact unless the routines for such contact were clear, remote communication discouraged joking because of potential misinterpretations over the phone, quiet moments during a conversation was also highlighted, something that was perceived as more difficult over the phone, at times they struggled to interpret messages (e.g., smileys) and that formulating a text could be both time- and energy-consuming, remote support challenged their cognitive functioning, where they risked not getting anything done because they forgot, had difficulties getting started, or were unable to implement new strategies  more difficult to stay focused, solve problems, and remain engaged when nobody was there to keep them focused on the activity, presence of another person could make a significant difference to a support meeting making it clearer and ‘more real’ and providing a ‘positive influence’ that could promote action |
|  | Support workers and service users | need for increased knowledge and skills in digital technology among the support workers, digital contact may be stressful and anxiety-provoking for some young adults, digital contact may feel less personal and create a sense of distance, human contact has an energy or power that might be missing in digital service encounters. They emphasized the “emotional presence” of in-person meetings as a factor that may help reduce young adults’ stress and provide a sense of security, need for in-person meetings to promote social connection and reduce social isolation, communication remotely was a complex task and non-verbal cues were less clear, more practical tasks were described as better suited for on-site support (e.g., sensitive financial issues and important meetings). |
| Participants with alcohol use disorder who were enrolled in the study “Blending internet treatment into conventional face-to-face treatment for alcohol use disorder (Blend-A)” but had not used intervention [46] | Patients with Alcohol use disorder (AUD) toward use of iCBT | noncompletion of an internet-based intervention to be dissatisfaction with the intervention itself, for example, that it was too time consuming or demanding and did not meet personal needs, lack of recognition in the content of the intervention, too much text and repetition, too little (meaningful) support or feedback, lack of contact with a therapist, and lack of guidance |
|  | Persons with AUD who declined use of bCBT | Preference toward face-to-face only |
|  | Participants who enrolled in the Blend-A study but declined to use bCBT (2/11 users) | had limited digital skills and competence to receive treatment via internet, believed they did not understand, felt “terrible” at it, |
|  | 1 participant who enrolled but declined use of bCBT, was a former internet user and had used technologies at work | Lack of digital skills |
|  | 9/11 participants, women with AUD in another study presented with eHealth treatment | Preferred in-person treatment, felt the need to attend sessions themselves in person to keep them accountable toward consistent treatment, believed in-person treatment is the only way to manage their emotional response to treatment, believed internet treatment to be impersonal and lacked ability to communicate instantly and effectively (verbally and via body language), iCBT would decrease their motivation toward treatment, face to face offers more privacy, doubted quality of feedback regarding digital or asynchronous feedback |
|  | Participants of another study presented with web-based treatment | Believed the therapist would neglect their needs |
| Persons 65+ years old with and without mild cognitive impairment at a local day center [47] | Senior adults (compared to general population) | Disparities in access to and utilization of digital technologies (internet and modern devices like smartphones, tablets, and smart speakers), also associated with age (not growing up with current digital technologies), socioeconomic status related to barriers obtaining necessary equipment and internet access, negative perceptions about new technological services and devices which can compromise some of the benefits expected from the introduction of technology in healthcare settings and benefits related to social interaction |
|  | 88% of all participants (related to smart speaker) | Never used a tablet or email |
|  | 84% of all participants (related to smart speaker) | Never used social networks |
|  | 80% of all participants (related to smart speaker) | had never used a computer |
|  | 72% of all participants (related to smart speaker) | Never used the internet |
|  | 80% of all participants (prior to smart speaker use) | Previously unaware of smart speaker technologies |
|  | 44% of all participants (prior to smart speaker use) | Never used smart speakers but were aware of them |
|  | 16% of all participants (prior to smart speaker use) | Believed smart speakers were not useful |
|  | 2 participants (after smart speaker use) | Smart speaker technology was not useful, 1 of whom did not enjoy the technology |
|  | 28% (related to smart speaker use) | Preferred face to face interactions |
|  | 92% of all participants (related to serious games via tablet) | Never used similar tools before, unaware of how they worked |
|  | 16% of all participants (related to serious games via tablet) | Aware but chose not to use serious games on tablets |
|  | Most participants | Preferred smart speaker over tablet and serious games (usefulness, ease of use, or enjoyment) |
|  | Health professionals | Time allotted to response to automatic voiced agents is too short (people need more time given cognitive decline), smart speakers are not adapted for users with hearing deficits |
| Patients without severe cognitive difficulties admitted to a neurological department [48] | Older population in Denmark | Less use of digital services |
|  | 5% of people aged 65 to 75 years and 18% of those aged 75 to 89 years in Denmark | Never used the internet |
|  | older citizens who are socially disadvantaged | Digitization of the healthcare system contributes to marginalization |
|  | few participants who did not own the necessary digital tools, those who did not use digital tools | did not have the skills needed to use these tools, lack of material resources was tied particularly to a lack of knowledge and know-how (where to buy, how to set up and connect to internet), feelings of being left out of society, digital systems seemed to provide unfair treatment based on something outside of their own power, physical medical conditions prevent use |
|  | Some participants who had access to a computer, smartphone, and tablet and knew how to use these tools, and participants with physical, cognitive, and communicative challenges | had to obtain help when setting up hardware, updating software, or getting a new device, needed social resources, Symptoms of health related to motor skills and mobility |
|  | Participants with cognitive and communicative inabilities and those experiencing health issues that directly affect memory and cognitive abilities | difficultly using digital tools, and this might not always be clear until the individual tries to use a device’s more complex functions, Difficulty remembering passwords, uncertainty about how to use the technology |
|  | Some participants | amount of extra time and energy that the participants had to spend on digital services, after they were discharged from the hospital, they did not use the computer anymore because it was too *difficult* and too *exhausting*, which contributed to them *giving up*. Using digital tools already demanded a certain amount of concentration and awareness, and some diseases and health conditions affected these abilities further, also required people to learn things repeatedly due to memory challenges, frustration with technology moving “too fast”, feelings of alienation, anxiety over using technology, fear (especially about making mistakes when using the digital healthcare system and fears of digital services replacing human interaction) and “stupidity”, required courage to try to use them, negative previous experiences (also related to physical conditions), uncertainty about effectiveness of their use, beliefs that it was “too late for them”/ learning to use was hopeless, unaware of options for digital services, |
| Those with South Asian ethnicity who had or were at risk of diabetes and/or heart disease, with access to suitable devices [49] | South Asian populations in the United Kingdom related to SMS text messaging (another study) | Face digital inequalities, challenges related to acceptability and design, lack of awareness and the process of downloading and setting up DHIs |
|  | [no group specified] | 25% of all apps are estimated to be uninstalled after only one use, barriers related to language, literacy, and digital access |
|  | 5/18 participants | did not use DHIs beyond receiving SMS text messages as part of appointment or other reminders, were unable to complete intervention tasks |
|  | participants who had limited or no knowledge of digital health | lack of digital skills, fear, and previous negative experiences such as scams, viruses, or errors leading to loss of money |
|  | Some participants | not confident digitally and needed assistance, concerned about future use their support network, e.g. family, moved away,  lack of clarity or instructions about what to do when setting up or using an app for the first time, voice recognition not being good at picking up accented English, a lack of culturally specific information, and apps not always returning relevant results, barriers to initial and continued use of digital for health purposes included the affordability of DHIs, complexity of sign-ins to maintain security, and suitability to manage their health needs, reasons for stopping app setup led to people choosing not to use the app (malfunctions (such as the app freezing or not syncing), needing to repeat log-in and administrative tasks, or a lack of integration with other apps), unknowns about data sharing and privacy (e.g. A lack of clear and specific information about permissions led to concerns such as that agreeing to one set of permissions might give access to their data for another purpose), felt that they had limited benefit for themselves, time-consuming nature of entering exercise or diet data, or reacting to notifications, was another reason that people did not feel DHIs could meet their current needs, maintaining behavior change was identified as being challenging |
|  | 71 year old nonuser | Unable to begin the task |
|  | 2 participants | Lacked skills to continue task |
|  | Some participants described being willing to use DHIs | not aware that they existed, needed more information about their function, or wanted recommendations from trusted sources such as the NHS |
|  | Participants who use digital tools in other aspects of their life | limiting their digital use for health self-management was a choice, preference toward directly speaking to someone, found no benefit to app use over existing actions to manage their health condition; or considered current service provision to be satisfactory and did not need to use digital to engage with health care providers, The need to set up health apps in a secure fashion, such as remembering passwords, entering a lot of information, or setting up other security features, caused some people to pause the setup process to seek help or terminate the activity |
|  | 2 participants who chose to complete intervention tasks on desktop computers | apps as having poor usability for individuals with complex needs (e.g., cannot scroll and select all medications for reordering, or the buttons being too small on a smartphone, making entering data “tricky”), |
|  | 5 participants who did not complete installation of the app | Concerns about privacy and permissions, Difficulty with access to the terms and conditions through an external link; the length of terms and conditions and the technical language used; and not being sure of what they should be looking for were all highlighted as specific worries, did not necessarily understand the purpose and requirement for permissions |
|  | few participants | Anxiety around health caused by searching for health information (“*it was like my addiction*”), constant monitoring, competing with others, not meeting goals, or potential inaccuracy of readings led to discontinued or less use, Avoided looking at historical data as it reflected their decreased mobility, preferred to make practical decisions based on experience rather than relying on generic advice, unable to adapt to advice, lack of explanation about how they work and how to set achievable goals (related to activity trackers), limited ways in which they interacted with technology |
|  | 2 participants | preference for not taking a phone to track exercise due to concerns about theft or loss |
|  | Most participants | most participants did not know where and how to find and select the apps relevant to their needs |
|  | participants who had sufficiently embedded the required behavior change to achieve stable management of their cardiometabolic condition | DHIs were identified as having a natural endpoint or a reduced role |

**Table 2. Technology-related requirements for inclusion and technology described in each study, by recruited group**

| **Groups recruited [REF]** | **Technology requirements for inclusion** | **Technology investigated by study** |
| --- | --- | --- |
| Patients with distressing paranoia from community mental health services [1] | N/A | SlowMo mobile app |
| interviewees (users and non-users) through their participation in the Oxford Internet Survey and in public places [2] | N/A | health information on the internet |
| Patients with T2D [3] | N/A | Digital health intervention integrated into routine care |
| Patients registered with the CIE patient-portal [4] | had logged in at least once during the study period, had to follow link to survey | Patient portal |
| Respondents from Center data’s LISSPANEL, a representative population [5] | Had to own smart device | Mobile tech |
| Representative of resident adult population (20+ years) from the Population Register Center [6, 7] | Participation required online survey response | Online health and social care services, internet, computer |
| individuals who had engaged with institutions providing homeless services and family shelters [8] | N/A | Internet for health purposes, apps and digital health |
| Case manager of patients with psychotic disorders and functional impairments from a specialized referral outpatient unit [9] | Completed digital survey | Access to smartphones and digital-ID |
| Pregnant women with legal residence from antenatal care centers [10] | N/A | eHealth literacy, health literacy, use of e-Boks (national digital mail system) |
| Home-dwelling persons with dementia, from memory clinics at local hospitals, municipal memory teams, and through advertisements in general media, and their informal caregivers [11] | N/A | Access to assistive technology and telecare (ATT), e.g. Social alarms or fall sensors, or just assistive technology (AT), e.g. Memory, tracking, or communication technology |
| Four generational cohorts from companies and retirement homes [12] | Had to complete online questionnaire | New communication technology (NCTs), eHeals (self-perceived assessment of health related digital skills), eHealth literacy (2nd and 3rd level) |
| Persons with cancer from oncology or urology outpatient clinics [13] | N/A | health related internet use (health online support questionnaire HOSQ), eHealth service (My Healthcare Contacts), health apps |
| Older adults and oldest aged 75-99 [14] | N/A | internet use |
| Older Russian-speaking migrants who are active internet users, and third-sector representatives who assist migrants with digital service use [15] | N/A | internet and digital public health and welfare services, Kela, the Social Insurance Institution of Finland |
| Surgical patients from public day surgery units [16] | with internet-connecting mobile phone | Mobile Phone in Recovery after Ambulatory Surgery’ (MIRAS) which evaluated a web-based mobile phone system |
| Persons with clinical epilepsy who were admitted for pre-surgical evaluation at the epilepsy monitoring unit [17] | N/A | wrist-worn seizure tracker |
| Persons from closed and open prisons [18] | N/A | Digital social and health care services |
| People with vulnerabilities in mental health from organizations providing community-based mental health rehabilitation, support, and sheltered employment services [19] | N/A | Digital social and health care services |
| Adults from patient organizations, online from hospitals and health clinics in multiple countries [20] | N/A | Patient health records |
| Random sample representative of the population ID’d via population registry [21] | N/A | Online health and social care services |
| Expert developers and distributors of digital therapeutics from digital health databases and press searches [22] | Experience with patient access to digital therapeutics (DTx) | DTx (digital therapeutics) |
| Non-emergency surgery patients from outpatient department [23] | N/A | Computers, smartphones/cell phones and broadband internet in general, web-based info for health, fitness devices, health apps (in general and: medication reminder, monitoring vital signs, appointment maker, health insurance company info/access to patient data, web-based consultations or video services, EHR |
| members of a Diabetes organization with T1 and/or T2D [24] | N/A |  |
| Immigrant women with a Pakistani family background, and persons with experience and/or positions of relevance [25] | N/A | mammographic screenings, BreastScreen Norway’s website |
| Elderly patients (65–80 years) with a chronic disease from primary health care centers [26] | N/A | general eHealth, video consults, PHCC’s website |
| Persons currently experiencing clinically significant symptoms of major depressive disorder in multiple countries [27] | N/A | mHealth for remote measurement and management, mHealth systems that require direct interaction with an app, wearable devices |
| Persons 50+ years who suffer from chronic pain [28] | some recruited from a previous eHealth research study | E-health in general, computers |
| Elderly Russian-speaking migrants who were not digitally proficient from workshops that included digital services in their curriculum [29] | N/A | Digital health and social services |
| Patients from a large secondary mental health provider who were digitally excluded [30] | screening questionnaire determined digital exclusion | Internet-abled technology |
| Community-dwelling healthy older adults [31] | N/A | Tablet Computers and technology in general |
| People with severe mental illness from community organizations who are digitally excluded [32] | screening questionnaire determined digital exclusion | Technology in general, social media |
| Staff, patients people with long-term conditions and carers in the implementation and evaluation component of study about digital patient feedback system [33] | Must have used the tested system | A kiosk for digital feedback, internet |
| Service users with intellectual disability [34] | who had previously taken part in a computerized therapy skills training session | Computers in psychological therapy |
| Elderly persons (65+) living in urban countryside (rural areas) from senior citizen meetings (who had not used digital health consultations before) [35] | who had not used digital health consultations before | Digital health services/consultations: Video and chat consultations used in primary care offered by private and public care providers |
| Patients with colorectal cancer after hospital discharge, their informal caregivers, and HCPs [36] | N/A | Information and communication technology |
| Adults from database of households currently participating in a project [37] | N/A | Use of existing digital technology such as apps, wearables, video calling software, social networks, virtual assistants) and the internet in general |
| Persons with T2D diabetes who are disadvantaged sub-segment of the community, requiring utmost care and consideration, including presence of comorbidity and risky lifestyle behaviors, have poor diabetes management, have low education and health literacy, from outpatient clinic [38] | N/A | potential of making an intervention: tailored digital intervention to improve self-management, internet |
| Adults (70+ years old) with chronic kidney disease who lived in areas of deprivation [39] | N/A | digital health technologies |
| People with an without intellectual, visual, calculation, language, impairments [40] | Responding “Not applicable” related to the following variables in the survey: booking appointments online, digital identification, website for the Swedish Social Insurance Agency (SSIA) and Swedish national web-portal for health information and eHealth services | eHealth |
| People receiving long-term dermatology follow-up [41] | N/A | Health technology |
| People (18+ years old) who had and had not previously used NHS 111 online [42] | Use of SMS text message mail out to receive the survey and completed the survey by following web-based link or on a computer tablet in the waiting room | NHS 111 Online Urgent Care Service |
| Post-discharge stroke survivors with aphasia and milder cognitive and physical disabilities [43] | Could handle computers | Electronic Care and Rehabilitation Planning Tool |
| Frail older adults who had completed most or all of the +AGIL Barcelona, “A community programme of integrated care for frail older adults”, and their providers [44] | Completed at least half of the +AGIL Barcelona program (including digital component - Vivifrail app) | Vivifrail app as part of the +AGIL Barcelona program |
| Young adults with a diagnosis of autism and/or ADHD who use support services, and their support workers [45] | N/A | remote elements in daily living support |
| Participants in the Blend-A study who did not engage in bCBT for alcohol use disorder [46] | Did not engage in the blended-CBT program for AUD | Blended-A, face-to-face and remote CBT program |
| Persons 65+ years old with and without mild cognitive impairment at a local day center [47] | Exclusion based on persons declaring technological aversion or phobia | smart speakers vs. serious gaming on tablets as cognitive assessment instruments |
| Patients without severe cognitive difficulties admitted to a neurological department [48] | N/A | digital health services and digital tools |
| Those with South Asian ethnicity who had or were at risk of diabetes and/or heart disease, with access to suitable devices [49] | Had access to a suitable device (smartphone, tablet, or laptop). Participants were offered WiFi access to facilitate downloading the app, if required. | digital health interventions (DHIs) for prevention and management of cardiometabolic diseases (CMD) |

**Table 3. Themes and sub-themes of reasons for experiencing the digital divide, grouped by level of digital divide.**

| **Theme:**  **“Level”/**  **Area of digital divide** | **Primary sub-themes:**  **Categories** | **Secondary sub-themes:**  **Factors** |
| --- | --- | --- |
| **1** | **Infrastructure (societal and healthcare system)** | 27 Accessibility or availability (including access to internet) [1-3, 6-8, 10-13, 15, 20, 21, 23, 25, 28, 33, 37-41, 43-47, 49] |
|  |  | Research activities [3, 6, 7, 15, 37, 43] |
|  |  | Resources and support [6, 7, 14, 19, 20, 25, 26, 28, 32, 34, 37, 39, 40, 48] |
|  | **Personal** | Awareness [6-8, 19, 21, 23, 25-27, 32, 35, 39, 44, 47-49] |
|  |  | Competency and/or capacity [19-21, 23-25, 41, 42, 44] |
|  |  | Health status or wellbeing [15, 35] |
|  |  | Socio-demographic/economic deprivation [3, 11, 14, 15, 21, 28, 33, 39, 47] |
|  |  | Social relationships (marginalization, discrimination) [28, 38] |
|  |  | Engagement (e.g. previous experience, ownership) [1, 23, 25-27, 31, 39, 47] |
|  | **Technology** | Availability (of mobile devices) [3, 12, 45] |
|  |  | Cost of technology (prohibitive) [4, 11, 33, 34, 37, 45, 49] |
|  | **Infrastructure (societal and healthcare system)** | Resources and support [5, 15, 16, 19, 21, 35, 39, 44, 45, 47-49] |
|  | **Personal** | Acceptance (e.g. disinterest in, distrust, motivation, choice) [1, 3, 6-8, 10, 12, 15-19, 21, 23, 25-30, 32-36, 38-40, 44-49] |
|  |  | Competency and/or capacity (e.g. knowledge and skills for use, competing responsibilities, negative beliefs about abilities,) [1, 6-9, 16, 17, 19-28, 32-41, 43-46, 48, 49] |
|  |  | Health status or wellbeing (e.g. age, cognitive or physical decline or limitations) [8, 9, 11-13, 21, 26-28, 32-40, 44, 45, 47-49] |
|  |  | Use of or relationship with healthcare services [6-8, 17, 21, 25, 27, 28, 31, 35, 36, 38, 39, 44-47, 49] |
|  |  | Socio-demographic/economic deprivation (language, occupation, immigrant status)[10, 12, 13, 19, 21, 27, 28, 34, 35, 37, 49] |
|  |  | Social relationships (e.g. support, marginalization, isolation, stigma etc.) [16, 20, 21, 28, 32, 38, 39, 45, 47] |
|  |  | Engagement (not optimal use, discontinued use) [1, 3, 6-8, 10, 16-18, 20-24, 26-28, 30-33, 35, 37-42, 44-49] |
|  | **Providers or healthcare personnel** | Acceptance [22, 33, 45] |
|  |  | Competency and/or capacity (Healthcare providers) [33, 34, 37, 45] |
|  |  | Cost of technology [34] |
|  | **Technology** | Quality [23, 34, 35, 37, 45-47] |
|  |  | Availability (want to use but technology not available) [11, 33] |
|  |  | Features, overall quality (affecting relevance to users) [11, 18, 21, 23, 33-37, 39, 43-49] |
|  |  | Privacy/security concerns [18, 21, 28, 29, 33, 37, 45, 46, 49] |
|  |  | Engagement (usability, amount of time or effort to use) [1, 6, 7, 9, 11, 25, 26, 28, 33, 35-37, 39, 40, 44, 45, 47-49] |
|  |  | Training, guidance or support for use [6, 7, 19, 20, 32, 35, 38, 43, 48, 49] |
| **3** | **Healthcare and healthcare system** | Quality [28, 33, 34, 38, 45] |
|  | **Personal** | Competency and/or capacity [21, 23, 39, 44-49] |
|  |  | Health and wellness outcomes [12, 23, 25, 27, 34, 36, 37, 39, 45, 46, 48, 49] |
|  |  | Non-health/socio economic outcomes (e.g. employment) [12, 21, 25, 38, 39, 44, 47-49] |
|  |  | Relationship with healthcare services [27, 28, 33, 34, 37, 39, 44-47] |
|  |  | Societal relationships (discrimination, stigma, social isolation, connectedness, reliance on others) [6, 7, 12, 15, 16, 18, 34, 36, 38, 44, 45, 47-49] |
|  |  | Engagement (lower engagement as outcome, or fear of misuse as outcome, or dependence on tech) [30, 32, 33, 36, 37, 49] |
|  |  | Accessibility of data by third parties [18, 21, 28, 29, 33, 37, 45, 46, 49] |
|  | **Technology** | Personal impact (e.g. intrusive, result in negative personal perceptions, general personal dangers, negative personal experiences) [6, 7, 13, 15-18, 25, 28, 32, 36-39, 45, 48, 49] |
|  |  | Quality [23, 45, 49] |

## References

1. Hardy A, Ward T, Emsley R, Greenwood K, Freeman D, Fowler D, et al. Bridging the digital divide in psychological therapies: observational study of engagement with the slowmo mobile app for paranoia in psychosis. JMIR Human Factors. 2022;9(3):e29725.

2. Powell J, Deetjen U. Characterizing the digital health citizen: mixed-methods study deriving a new typology. Journal of Medical Internet Research. 2019;21(3):e11279.

3. Poduval S, Ahmed S, Marston L, Hamilton F, Murray E. Crossing the digital divide in online self-management support: analysis of usage data from HeLP-diabetes. JMIR diabetes. 2018;3(4):e10925.

4. Neves AL, Smalley KR, Freise L, Harrison P, Darzi A, Mayer EK. Determinants of use of the care information exchange portal: cross-sectional study. Journal of Medical Internet Research. 2021;23(11):e23481.

5. Bol N, Helberger N, Weert JC. Differences in mobile health app use: a source of new digital inequalities? The Information Society. 2018;34(3):183-93.

6. Heponiemi T, Jormanainen V, Leemann L, Manderbacka K, Aalto A-M, Hyppönen H. Digital divide in perceived benefits of online health care and social welfare services: national cross-sectional survey study. Journal of medical Internet research. 2020;22(7):e17616.

7. Heponiemi T, Gluschkoff K, Leemann L, Manderbacka K, Aalto A-M, Hyppönen H. Digital inequality in Finland: access, skills and attitudes as social impact mediators. New Media & Society. 2023;25(9):2475-91.

8. Radó N, Girasek E, Békási S, Győrffy Z. Digital Technology Access and Health-Related Internet Use Among People Experiencing Homelessness in Hungary: Quantitative Survey. Journal of Medical Internet Research. 2022;24(10):e38729.

9. Holmberg C, Gremyr A, Karlsson V, Asztély K. Digitally excluded in a highly digitalized country: An investigation of Swedish outpatients with psychotic disorders and functional impairments. The European Journal of Psychiatry. 2022;36(3):217-21.

10. Villadsen SF, Hadi H, Ismail I, Osborne RH, Ekstrøm CT, Kayser L. ehealth literacy and health literacy among immigrants and their descendants compared with women of Danish origin: a cross-sectional study using a multidimensional approach among pregnant women. BMJ open. 2020;10(5).

11. Puaschitz NG, Jacobsen FF, Mannseth J, Angeles RC, Berge LI, Gedde MH, et al. Factors associated with access to assistive technology and telecare in home-dwelling people with dementia: baseline data from the LIVE@ Home. Path trial. BMC Medical Informatics and Decision Making. 2021;21(1):1-14.

12. Papp-Zipernovszky O, Horváth MD, Schulz PJ, Csabai M. Generation gaps in digital health literacy and their impact on health information seeking behavior and health empowerment in Hungary. Frontiers in public health. 2021;9:635943.

13. Mattsson S, Olsson EMG, Johansson B, Carlsson M. Health-related internet use in people with cancer: results from a cross-sectional study in two outpatient clinics in Sweden. Journal of medical Internet research. 2017;19(5):e163.

14. Quittschalle J, Stein J, Luppa M, Pabst A, Löbner M, Koenig H-H, et al. Internet use in old age: results of a German population-representative survey. Journal of medical Internet research. 2020;22(11):e15543.

15. Buchert U, Kemppainen L, Olakivi A, Wrede S, Kouvonen A. Is digitalisation of public health and social welfare services reinforcing social exclusion? The case of Russian-speaking older migrants in Finland. Critical Social Policy. 2023;43(3):375-400.

16. Poli A, Kelfve S, Berg K, Motel-Klingebiel A. Old-age diversity is underrepresented in digital health research: Findings from the evaluation of a mobile phone system for post-operative progress monitoring in Sweden. Ageing & Society. 2021:1-23.

17. Bruno E, Biondi A, Thorpe S, Richardson M, Consortium R-C. Patients self-mastery of wearable devices for seizure detection: a direct user-experience. Seizure. 2020;81:236-40.

18. Rantanen T, Järveläinen E, Leppälahti T. Self-efficacy and Use of Digital Health Care and Social Welfare Services Among Incarcerated People: Cross-sectional Survey Study. Journal of Medical Internet Research. 2022;24(5):e36799.

19. Tetri B, Juujärvi S. Self-efficacy, internet self-efficacy, and proxy efficacy as predictors of the use of digital social and health care services among mental health service users in Finland: A cross-sectional study. Psychology research and behavior management. 2022:291-303.

20. Paccoud I, Baumann M, Le Bihan E, Pétré B, Breinbauer M, Böhme P, et al. Socioeconomic and behavioural factors associated with access to and use of Personal Health Records. BMC medical informatics and decision making. 2021;21:1-11.

21. Rantanen T, Gluschkoff K, Silvennoinen P, Heponiemi T. The associations between mental health problems and attitudes toward web-based health and social care services: evidence from a Finnish population-based study. Journal of medical Internet research. 2021;23(9):e28066.

22. Dahlhausen F, Zinner M, Bieske L. There’s an app for that, but nobody’s using it: Insights on improving patient access and adherence to digital therapeutics in Germany. Digit Health. 2022.

23. Korn S, Böttcher MD, Busse TS, Kernebeck S, Breucha M, Ehlers J, et al. Use and Perception of Digital Health Technologies by Surgical Patients in Germany in the Pre–COVID-19 Era: Survey Study. JMIR Formative Research. 2022;6(5):e33985.

24. Hansen AH, Bradway M, Broz J, Claudi T, Henriksen Ø, Wangberg SC, et al. Inequalities in the use of eHealth between socioeconomic groups among patients with type 1 and type 2 diabetes: cross-sectional study. Journal of medical Internet research. 2019;21(5):e13615.

25. Bhargava S, Hofvind S, Moen K. Gender, letters, relatives, and God: mediating actors in mammographic screening among Pakistani women in Norway. Acta radiologica open. 2019;8(9):2058460119875015.

26. Nymberg VM, Bolmsjö BB, Wolff M, Calling S, Gerward S, Sandberg M. ‘Having to learn this so late in our lives…’Swedish elderly patients’ beliefs, experiences, attitudes and expectations of e-health in primary health care. Scandinavian journal of primary health care. 2019;37(1):41-52.

27. Simblett S, Matcham F, Siddi S, Bulgari V, di San Pietro CB, López JH, et al. Barriers to and facilitators of engagement with mHealth technology for remote measurement and management of depression: qualitative analysis. JMIR mHealth and uHealth. 2019;7(1):e11325.

28. O’Reilly PM, Harney OM, Hogan MJ, Mitchell C, McGuire BE, Slattery B. Chronic pain self-management in middle-aged and older adults: A collective intelligence approach to identifying barriers and user needs in eHealth interventions. Digital Health. 2022;8:20552076221105484.

29. Safarov N. Personal experiences of digital public services access and use: Older migrants’ digital choices. Technology in Society. 2021;66:101627.

30. Greer B, Robotham D, Simblett S, Curtis H, Griffiths H, Wykes T. Digital exclusion among mental health service users: qualitative investigation. Journal of medical Internet research. 2019;21(1):e11696.

31. Vaportzis E, Giatsi Clausen M, Gow AJ. Older adults perceptions of technology and barriers to interacting with tablet computers: a focus group study. Frontiers in psychology. 2017;8:1687.

32. Middle R, Welch L. Experiences of digital exclusion and the impact on health in people living with severe mental illness. Frontiers in Digital Health. 2022;4:1004547.

33. Ong BN, Sanders C. Exploring engagement with digital screens for collecting patient feedback in clinical waiting rooms: The role of touch and place. Health. 2021;25(4):454-74.

34. Vereenooghe L, Gega L, Langdon PE. Intellectual disability and computers in therapy: Views of service users and clinical psychologists. Cyberpsychology. 2017;11(1).

35. Landgren S, Cajander Å. Non-use of digital health consultations among Swedish elderly living in the countryside. Frontiers in Public Health. 2021:1323.

36. Husebø AML. Stakeholders’ Perspectives on eHealth Support in Colorectal Cancer Survivorship: Qualitative Interview Study. JMIR cancer. 2021;7(3):e28279.

37. Buckingham SA, Walker T, Morrissey K, team Sp. The feasibility and acceptability of digital technology for health and wellbeing in social housing residents in Cornwall: A qualitative scoping study. Digital Health. 2022;8:20552076221074124.

38. Mathiesen AS, Thomsen T, Jensen T, Schiøtz C, Langberg H, Egerod I. The influence of diabetes distress on digital interventions for diabetes management in vulnerable people with type 2 diabetes: a qualitative study of patient perspectives. Journal of Clinical & Translational Endocrinology. 2017;9:41-7.

39. Chadwick H, Laverty L, Finnigan R, Elias R, Farrington K, Caskey FJ, et al. Engagement With Digital Health Technologies Among Older People Living in Socially Deprived Areas: Qualitative Study of Influencing Factors. JMIR Formative Research. 2024;8:e60483.

40. Pettersson L, Johansson S, Demmelmaier I, Gustavsson C. Disability digital divide: survey of accessibility of eHealth services as perceived by people with and without impairment. BMC Public Health. 2023;23(1):181.

41. Ramjee S, Mohamedthani H, Patel AU, Goiriz R, Harwood CA, Osborne RH, et al. The Effect of Remote Digital Services on Health Care Inequalities Among People Under Long-Term Dermatology Follow-Up: Cross-Sectional Questionnaire Study. JMIR dermatology. 2023;6:e48981.

42. Turnbull J, Prichard J, MacLellan J, Pope C. eHealth Literacy and the Use of NHS 111 Online Urgent Care Service in England: Cross-Sectional Survey. Journal of Medical Internet Research. 2024;26:e50376.

43. Davoody N, Eghdam A, Koch S, Hägglund M. Evaluation of an Electronic Care and Rehabilitation Planning Tool With Stroke Survivors With Aphasia: Usability Study. JMIR Human Factors. 2023;10:e43861.

44. Canet-Vélez O, Solis-Navarro L, Sitjà-Rabert M, Pérez LM, Roca J, Soto-Bagaria L, et al. Experience, facilitators, and barriers to the implementation of a multicomponent programme in older people living in the community,+ AGIL Barcelona: A qualitative study. Frontiers in Public Health. 2023;11:1161883.

45. Löthberg M, Wirström E, Meyer J, Girdler S, Bölte S, Jonsson U. ‘If I Don’t Have My Support Worker in the Room…’: A Multi-perspective Mixed Methods Study of Remote Daily Living Support for Neurodivergent Young Adults. Journal of autism and developmental disorders. 2024:1-14.

46. Tarp K, Christiansen R, Bilberg R, Borkner S, Dalsgaard C, Folker MP, et al. Nonuse of Blended Web-Based and Face-To-Face Cognitive Behavioral Therapy for Alcohol Use Disorder: Qualitative Study. JMIR Formative Research. 2024;8(1):e45471.

47. Pacheco Lorenzo M, Lago Priego N, Fernández Iglesias MJ, Anido Rifón L, Otero-González I. Oral vs. touch interaction for cognitive assessment: acceptance and perceived usability by senior adults. Electronics. 2023;13(1):13.

48. Gybel Jensen C, Gybel Jensen F, Loft MI. Patients’ experiences with digitalization in the health care system: qualitative interview study. Journal of Medical Internet Research. 2024;26:e47278.

49. Ramasawmy M, Persson DR, Sunkersing D, Gill P, Khunti K, Poole L, et al. Uptake of Digital Health Interventions for Cardiometabolic Disease in British South Asian Individuals: Think Aloud Study. JMIR Human Factors. 2024;11(1):e57338.
